# Patient Benefits in the Context of Sepsis-Related AI-Based Clinical Decision Support Systems: Scoping Review

**DOI:** 10.2196/76772

**Published:** 2026-01-26

**Authors:** Pascal Raszke, Godwin Denk Giebel, Jürgen Wasem, Michael Adamzik, Hartmuth Nowak, Lars Palmowski, Philipp Heinz, Nina Timmesfeld, Marianne Tokic, Frank Martin Brunkhorst, Nikola Blase

**Affiliations:** 1 Institute for Health Care Management and Research, University of Duisburg-Essen, Thea-Leymann-Str. 9, Essen, 45127, Germany, 49 201 183 4395; 2 Department of Anesthesiology, Intensive Care Medicine and Pain Therapy, Ruhr University Bochum, Knappschaft Kliniken University Hospital Bochum, Bochum, Germany; 3 Department of Anesthesiology, Intensive Care Medicine and Pain Therapy, Center for Artificial Intelligence, Medical Informatics and Data Science, Ruhr-University Bochum, Knappschaft Kliniken University Hospital Bochum, Bochum, Germany; 4 Knappschaft Kliniken GmbH, Recklinghausen, Germany; 5 Department of Medical Informatics, Biometry and Epidemiology, Ruhr University Bochum, Bochum, Germany; 6 Institute of Infectious Diseases and Infection Control, Jena University Hospital, Jena, Germany

**Keywords:** medical informatics, artificial intelligence, machine learning, computational intelligence, clinical decision support systems, CDSS, decision support, sepsis, bloodstream infection

## Abstract

**Background:**

Global digitalization continues to advance, extending its influence into medicine and health care systems worldwide. In recent years, substantial advancements have been made in the research and development of artificial intelligence (AI), raising questions about its potential in medicine. The integration and application of AI in intensive care medicine, particularly in sepsis treatment, presents significant potential for advancing patient outcomes and enhancing patient-relevant benefits. However, a comprehensive and systematic overview of the full spectrum of patient-relevant benefits associated with AI-based clinical decision support systems (CDSS) remains lacking.

**Objective:**

This scoping review aimed to identify and categorize evidence on patient-relevant benefits of AI-based CDSS in sepsis care.

**Methods:**

Systematic research was conducted in 4 electronic databases: MEDLINE via PubMed, Embase, the ACM Digital Library, and IEEE Xplore. In addition, a comprehensive search on the websites of relevant international organizations, along with a citation search of the included articles, was conducted. Articles were included if they (1) focused on sepsis and (2) described patient-relevant benefits of AI-based CDSS. Articles published between January 1, 2008, and March 2, 2023, were considered for inclusion. Study selection was performed independently by 2 reviewers. The manuscript was drafted in accordance with the PRISMA-ScR (Preferred Reporting Items for Systematic Reviews and Meta-Analyses extension for Scoping Reviews) checklist. The analysis of the included articles was conducted using the program MAXQDA (VERBI Software GmbH), with systemization finalized in a consensus workshop.

**Results:**

A total of 3368 records were identified across the 4 databases, of which 24 met the inclusion criteria and were included in the scoping review. The additional search on international websites and in reference lists identified 6 more relevant articles, resulting in 30 included studies. Of these, 20 were quantitative, comprising 7 prospective and 13 retrospective designs. In addition, 1 qualitative study, 1 mixed methods study, 6 review articles, and 2 articles from institutional websites were included. Patient-relevant benefits were systematized in six main categories: (1) prediction, (2) earlier treatment and prioritization of high-risk patients, (3) individualized therapy, (4) improved patient outcomes (including improved Sequential Organ Failure Assessment score, reduced length of stay, and reduced mortality), (5) general improvements in care, and (6) reduced readmission rate.

**Conclusions:**

This scoping review underscores the potential of AI-based CDSS to positively impact patient-relevant benefits, particularly in sepsis care, where they demonstrate considerable promise for improving intensive care. However, the majority of the identified studies rely on retrospective database analyses. Future research should focus on validating these findings through prospective studies.

## Introduction

The treatment of infectious diseases has historically resulted in medical progress, exemplified by antibiotics and vaccines. Despite all medical advances, infections remain a major global cause of morbidity and mortality [1,2]. Sepsis, defined as “life-threatening organ dysfunction caused by a dysregulated host response to infection” [3], remains among the top contributors to worldwide mortality. It accounts for 30%‐50% of all hospital deaths in high-income countries, such as the United States [1], and approximately 11 million annual deaths worldwide [2]. Sepsis is a heterogeneous syndrome with variable phenotypes and outcomes. Thus, the interpretation of initial symptoms can be difficult for health care providers [3,4].

The effectiveness and accuracy of established rule-based scoring systems used for the assessment of patients in the intensive care unit (ICU), such as the systemic inflammatory response syndrome (SIRS) criteria, which were historically of importance, the sequential organ failure assessment (SOFA) score or the quickSOFA (qSOFA) score, the acute physiology and chronic health evaluation II (APACHE II) score, or the national early warning score 2 (NEWS2), is limited. This is partly because these scoring systems are not always specifically developed for sepsis patients and are therefore of limited use to health care providers in this context [5,6]. Nevertheless, timely identification and treatment are crucial to enhance patient outcomes [7-9], as untreated sepsis can progress to septic shock, exacerbating the patient’s condition [10] and leading to multiple organ failure, which carries an even higher mortality rate than sepsis itself [11].

This is where recent developments in artificial intelligence (AI) become particularly relevant, as they are considered to hold substantial potential for improving sepsis diagnostics. Especially machine learning (ML), a branch of AI, has the ability to rapidly analyze vast amounts of data, exceeding human capacity to process. By evaluating numerous data points, ML can derive conclusions and recognize correlations that a human health care provider would be incapable of identifying. This is why ML is well-suited as a technological foundation for clinical decision support systems (CDSS), particularly in the complex clinical picture of sepsis [3]. The use of ML in the development of CDSS can make the sepsis diagnosis more reliable, with the prospect of long-term improvements in patient outcomes. Machine learning algorithms (MLAs) demonstrated potential to enhance patient-relevant benefits in distinct studies. Documented benefits include, for example, reductions in sepsis-related mortality and the average hospital length of stay (LOS). Additionally, MLAs facilitate earlier interventions, such as the timely administration of antibiotics [12-14].

Despite the high clinical relevance of sepsis and significant advancements in both the availability of digital patient data and in the field of ML, the real-world application of AI-based CDSS remains negligible. The majority of these algorithms remain in the prototype phase, with deployment limited to a single hospital or a single hospital operator. This gap is highlighted by an analysis of the Food and Drug Administration’s database of medical devices using AI or ML. As of April 2025, none of the over 1000 listed products are specifically dedicated to intensive care [15], the medical field at the forefront of sepsis treatment. This illustrates the discrepancy between technological progress and its real-world implementation in the critical care environment. For AI-based CDSS to be successfully implemented in clinical practice, it is a necessary prerequisite that they demonstrate tangible added value. Accordingly, patient-relevant benefits should constitute a primary focus.

The research objective of the present study differs from those of previous scoping reviews on AI-based CDSS in sepsis care. Certain reviews focused specifically on neonatal [16] or pediatric [17] sepsis, whereas others concentrated on tasks for which MLAs were designed—such as risk assessment, treatment planning, or process support—and thus focused on the process of medical service delivery rather than on actual patient-relevant benefits [18] or on the actual design of the CDSS and its intended users [19]. Importantly, none of the aforementioned reviews [16-19] focused exclusively on patient-relevant benefits. Furthermore, several existing scoping reviews used narrow methodological approaches, for example, being restricted to a single ML method [17] or considering only antibiotic treatment of sepsis [16]. To the authors’ knowledge, no other scoping review has explicitly examined the patient-relevant benefits of AI-based CDSS in the context of sepsis while applying a broad and exploratory methodological approach, without restrictions regarding the ML methods used or the types of patient-relevant benefits assessed. Accordingly, the objective of the present study is to identify patient-relevant benefits of AI-based CDSS in sepsis care compared with the current standard of care, thereby addressing this research gap, as patient-relevant benefits constitute a meaningful benchmark for evaluating the value of any medical innovation. In this context, a taxonomy of benefits comprising 6 main categories has been developed.

This scoping review was conducted within the framework of the KI@work (User-Oriented Requirements for AI-Based Clinical Decision Support Systems) project, which is funded by the German Federal Joint Committee (funding code: 01VSF22050). The research project is led by the Institute for Health Care Management and Research at the University of Duisburg-Essen. Consortium partners include the Department of Anesthesiology, Intensive Care Medicine and Pain Therapy at the University Hospital Knappschaftskrankenhaus Bochum, the Knappschaft Kliniken GmbH, the Department of Medical Informatics, Biometry and Epidemiology at the Ruhr University Bochum and the German Sepsis Society. This scoping review addressed 2 additional research questions. However, to ensure a coherent presentation of the findings, this article focuses exclusively on patient-relevant benefits.

## Methods

### Overview

This scoping review is based on the methodology framework of the Joanna Briggs Manual for evidence synthesis [20], a further development of the work of Arksey and O’Malley [21] and Levac et al [22]. The review process followed the five stages originally described by Arksey and O’Malley: (1) identifying the research question, (2) identifying relevant studies, (3) study selection, (4) charting the data, and (5) collating, summarizing, and reporting the results [21]. The manuscript was prepared according to the PRISMA-ScR (Preferred Reporting Items for Systematic Reviews and Meta-Analyses extension for Scoping Reviews) checklist by Tricco et al [23] (Checklist 1). As scoping reviews encompass a broad range of study types in order to present a comprehensive overview of the research field [20-22], comparability between studies is limited. Consequently, no formal quality appraisal was conducted. Although no distinct protocol for the scoping review was published, the methodology was described in detail in a protocol for the overarching multimethod research project [24].

### Search Strategy

The development of the search strategy commenced with an initial limited search in MEDLINE via PubMed and Embase to identify relevant search terms. Subsequently, the identified terms were discussed in recurring team discussions within the consortium. In a third step, the consented search terms were combined into search queries.

The electronic databases MEDLINE via PubMed, Embase, as well as the ACM Digital Library and IEEE Xplore, were searched for relevant literature on March 2, 2023. The databases were selected to ensure that the interdisciplinary research question could be adequately addressed from both a medical and a computer science perspective. The search string was developed using the PCC (population=persons with or at risk of sepsis, concept=CDSS, and context=AI) framework. The MEDLINE via PubMed search string was quality-assured by the chief librarian at the library of the University Medical Centre Essen before the database search was conducted. The other 3 search strings were developed based on the same quality assurance principles as the MEDLINE via PubMed search query. The individual search terms were limited to occurrences in title, abstract, and keyword searches but were supplemented by indexing terms (MeSH and Emtree) and truncations. The final search strategies for each database can be found in Multimedia Appendices 1-4.

In agreement with ML experts (NT, HN), the search was limited to articles published in the last 15 years. Further explanation for the time restriction is provided in the discussion of this article. The search was restricted to English and German. In cases of missing full texts, the interlibrary loan service of the University of Duisburg-Essen was used. If that approach was not successful, the reviewers contacted the respective authors of the papers of interest. The identified citations were imported into the reference management program Endnote 20 (Clarivate Analytics).

In addition to the systematic search of electronic databases, a structured search for gray literature (eg, working papers and guidelines) from various governmental and nongovernmental stakeholders was conducted via their websites. The selection of countries included in the search was based on the results of the Bertelsmann #SmartHealthSystem study, which examined the degree of digitalization of various health care systems in 2018. It was assumed that the prospect of identifying information on AI-based CDSS would be particularly high in countries with highly digitalized health care systems. According to the Bertelsmann study, this applies to the health care systems of Canada, Denmark, Estonia, Israel, and Spain. In addition, 3 large economies—Germany, the United Kingdom, and the United States—were included in the structured research. Alongside institutional websites from these countries, websites of relevant international stakeholders were also examined. These included the World Health Organization (WHO) and the Organisation for Economic Co-operation and Development (OECD), as well as websites of international sepsis, intensive care, and medical informatics associations. Further information about included websites can be found in Multimedia Appendix 5. To supplement further evidence, reference lists of articles identified through the systematic and structured search were screened, and the cited articles were subsequently assessed for eligibility. If eligible, the referenced articles were included in the scoping review.

### Eligibility Criteria

Exploratory research and internal discussions contributed to the development of inclusion and exclusion criteria, which were refined iteratively during the initial stages of the research process. The search strategy was designed to address 3 different research questions. Studies were considered for inclusion if they described (1) patient-relevant benefits of AI-based CDSS in the context of sepsis as well as (2) problems in their development, implementation, or application, or (3) suggestions for improving these processes. Patient-relevant benefits were identified entirely exploratively and categorized independently of existing frameworks, allowing AI-based CDSS benefits to be classified without reliance on established definitions or patient-relevant endpoints. This approach provides a comprehensive and complete overview of the potential benefits of this emerging technology, without constraining the findings of this paper to predefined frameworks and definitions. Patient-relevant benefits were defined as the positive impact of an intervention on patients, irrespective of whether these comprise general qualitative observations or specific, measurable quantitative endpoints. Specific inclusion and exclusion criteria were developed for each research question to ensure a tailored approach to the unique scope of each question. AI was defined as ML-based algorithms that operate as a “black box” for the user (physician or caregiver), meaning their output is not directly interpretable for health care providers. Consequently, all ML-based technologies developed through data-driven training and sufficiently complex to preclude full comprehension by the user were eligible for inclusion. In contrast, rule-based algorithms, such as those relying on SIRS or SOFA criteria, did not meet this definition and were therefore excluded in this review. Moreover, earlier diagnosis facilitated by AI-based CDSS was not considered a patient-relevant benefit, as earlier diagnosis itself has no impact on patient outcomes. It is the interventions that follow an earlier diagnosis—such as increased attention by health care providers to patients developing sepsis or earlier initiation of treatment—that positively influence patient-relevant benefits. Accordingly, these parameters are pertinent to the scope of this review. Articles were selected regardless of the research method used. The inclusion criteria are presented in Textbox 1.

Exclusion criteria for this review were not answering the research question, an exclusively technical description of the algorithms developed, or exclusively mathematical approaches not providing evidence for patient-relevant benefit. In addition, articles were excluded if they focused only on the evaluation of binary classifiers such as sensitivity, specificity, positive predictive value, or negative predictive value, as the superiority of AI-based algorithms over rule-based scores was considered a prerequisite for such systems. AI-based CDSS developed exclusively for neonates and/or children or for animals were also not included, because (1) the treatment of neonatal or pediatric sepsis patients differs significantly from the treatment of adult patients [25-27] and (2) the focus of the study is on human sepsis. Articles published before 2008 were also excluded, as were those written in languages other than English or German. Research protocols, conference abstracts, letters to the editor, and articles that were only expressions of opinions were also excluded. The exclusion criteria are listed in Textbox 1.

Textbox 1. **Inclusion and exclusion criteria.**

**Inclusion criteria**

- Articles focusing on sepsis and
- Involving AI-based CDSS, that
  - Describe patient-relevant benefits, or
  - Describe problems with development, implementation, or application, or
  - Describe strategies for success

**Exclusion criteria**

- Exclusively technical description of systems, or
- Focus on description of the evaluation of binary classifiers, or
- Articles describing AI-based CDSS for neonates and children or animals, or
- Not addressing any of the research questions in more detail, or
- Research protocols, conference abstracts, theses, letters to the editor, or expression of opinions, or
- Article published before 2008, or
- Language other than English or German

### Evidence Screening, Selection, and Data Extraction

After identification and deletion of duplicates, title and abstract screening was conducted independently by 2 reviewers (PR and GDG) to decide whether an article was eligible for full-text screening. In a second step, the same 2 reviewers conducted a full-text screening of the included articles against the inclusion and exclusion criteria. In case of disagreement between the 2 reviewers during step 2 of the screening process, other members of the study team (NB, HN, and NT) were involved to decide whether an article was eligible for inclusion.

MAXQDA (VERBI Software GmbH) software was used to identify and tag relevant content in the included articles and to precategorize the patient-relevant benefit categories (PR) using an inductive coding approach. The preliminary categories were discussed and further refined in an in-person workshop based on the affinity mapping technique (PR, NB, and GDG). For this purpose, all relevant text passages were printed as snippets and physically assigned to the respective preliminary categories before being refined and finalized during the workshop. Each assignment was discussed in detail until full consensus among all 3 team members was reached. The results of the workshop were subsequently digitalized in Microsoft Excel. In addition to the patient-relevant benefits of AI-based CDSS, metadata, such as participating authors, year of publication, country of study, database for MLA, or study type, were extracted and summarized (see Multimedia Appendix 6).

### Analysis and Presentation of Results

The results of the included studies were summarized descriptively, and analysis was conducted to derive implications for policy, practice, and research. The patient-relevant benefits were grouped into 6 main categories. The main categories were presented in tabular form in an Excel file and diagrammatically. The patient-relevant benefit categories are presented chronologically in Multimedia Appendix 7.

## Results

### Selection of Sources of Evidence

#### Selection Process

**Figure 1.**
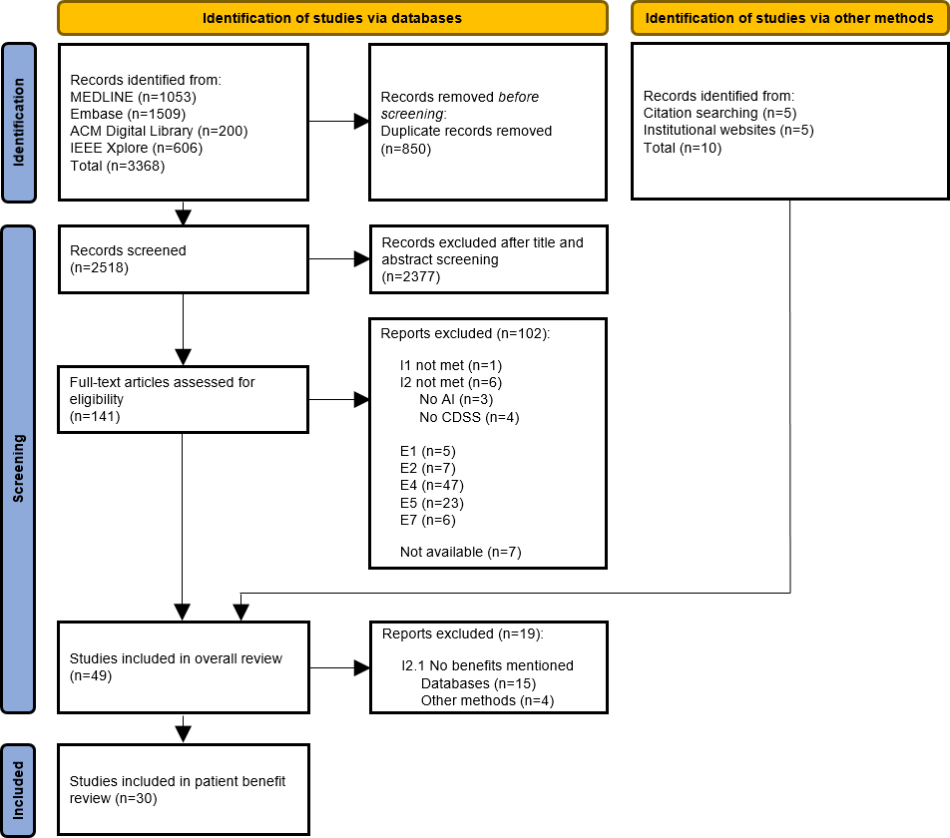
Flow diagram illustrating the selection process of evidence. CDSS: clinical decision support system.

In the systematic search, a total of 3368 titles and abstracts were retrieved. After removing 850 duplicates, 2518 articles remained for screening (Figure 1). Of these, 141 articles were screened for full text, and 39 met the inclusion criteria. Among these, 24 provided statements on patient-relevant benefits [28-51]. In addition, reference lists of the articles identified through the systematic search were analyzed, resulting in the identification of 5 additional articles, 2 of which reported information on patient-relevant benefits [52,53]. A complementary search of institutional websites led to the inclusion of 5 additional articles, 4 of which contained relevant information on patient-relevant benefits [54-57]. In total, 30 articles were included in the scoping review about patient-relevant benefits. The full-text screening process, including a detailed account of the reasons for exclusion, is presented in Multimedia Appendix 8.

#### Included Studies

Of the 30 articles included, 16 originated from North America, all of which are from the United States (53.3%) [28,30-32,34,36-38,42,43,45,49,52,53,56,57]. Seven articles stem from Europe (23.3%); 3 from the Netherlands (10%) [46,48,50], 2 from Spain (6.6%) [33,44], 1 from Austria (3.3%) [29], and 1 from the United Kingdom (3.3%) [39]. Five articles are from Asia (16.7%), including 2 each from China [41,51] and Taiwan [40,54] (6.6% each), and 1 from Singapore (3.3%) [35]. There is 1 article from Australia (3.3%) [55] and 1 article from South America (Brazil) (3.3%) [47].

The study designs used in the included articles cover a wide range. Overall, 20 quantitative articles were identified. Of these, 7 used a prospective study design, of which 2 are multicenter studies [28,31] and 5 are single-center studies [34,43,49,52,54]. Thirteen of the quantitative studies used a retrospective approach, comprising 6 research database studies [29,30,39,40,46,53] and 7 electronic health record database studies [33,35,41,42,45,47,50]. In addition to the quantitative articles, 1 article used a qualitative approach [37] and another applied a mixed methods approach [36]. Additionally, 6 review articles [32,38,44,48,51,55] and 2 articles from news sections of institutional websites were identified [56,57]. All articles are listed in Multimedia Appendix 6.

### Synthesis of Results

**Figure 2.**
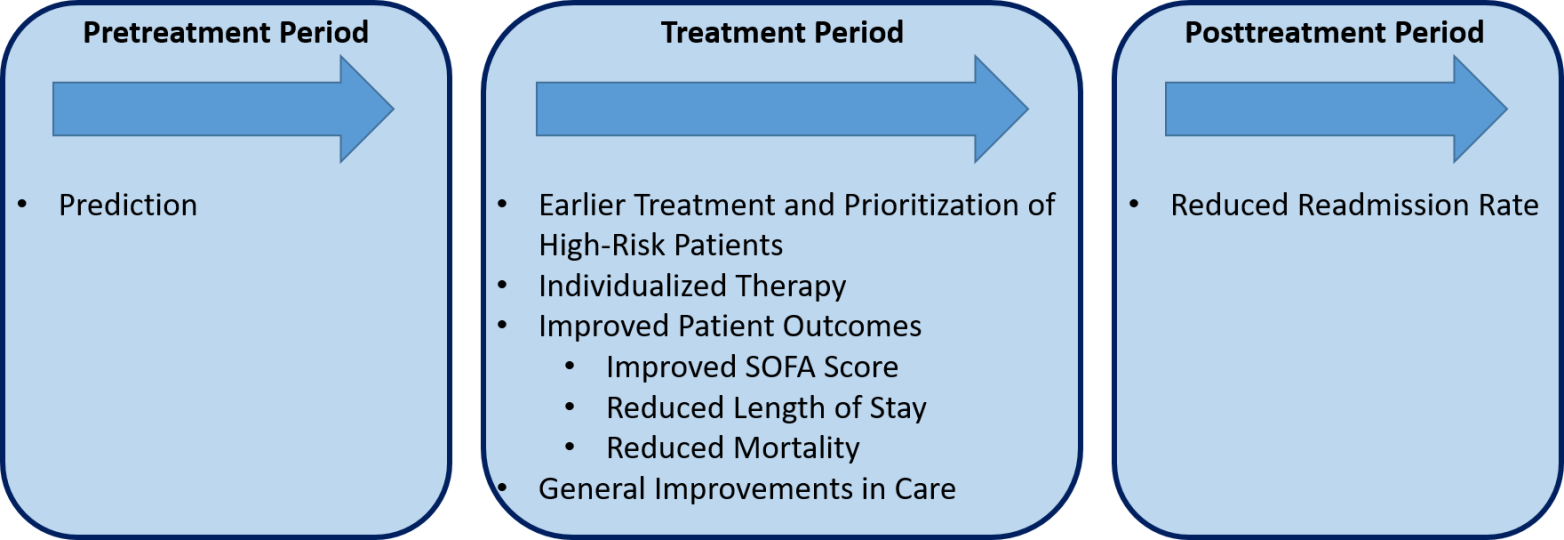
Patient benefit categories related to artificial intelligence-based clinical decision support systems.

In total, 6 main categories of patient-relevant benefit were identified. The 6 main categories identified reflect the patient pathway from pretreatment to posttreatment period. They include (1) prediction, (2) earlier treatment and prioritization of high-risk patients, (3) individualized therapy (which encompasses patient-centered care), (4) improved patient outcomes (which includes improved SOFA score, reduced length of stay, and reduced mortality), (5) general improvements in care, and (6) reduced readmission rate (see Figure 2). Multimedia Appendix 7 gives a detailed overview of the benefit categories addressed in each study.

### Pretreatment Period (Prediction)

Prediction of sepsis, septic shock, or sepsis-related organ dysfunction was addressed in 12 articles [31,33,35,37,38,40,42,45,48,51,53,56], comprising 7 quantitative studies (1 prospective [31] and 6 retrospective [33,35,40,42,45,53]), 1 qualitative study [37], 3 reviews [38,48,51], and 1 institutional news report [56]. The MLAs identified in this review indicate predictive capacity [48], which may be further optimized through algorithm fine-tuning [45]. Findings suggest that these models may predict sepsis between 4 and 48 hours prior to its onset [35,38,40,51,56], even before significant changes in vital or laboratory parameters become apparent [40]. MLAs were reported to support the identification of appropriate preventive measures [33]. Such predictions may have the potential to improve patient outcomes by providing timely warning of sepsis onset [31,37]. Beyond sepsis, the studies also reported the prediction of septic shock, with MLA predictions occurring between 4 and 7 hours before the onset of septic shock [42,51]. Compared with traditional rule-based routine screening protocols, predictive MLAs demonstrated superior early warning performance, identifying 58.6% more patients before organ dysfunction [53] and potentially contributing to a reduction in septic shock incidence [38]. Moreover, MLAs were shown to predict sepsis-related organ dysfunction approximately 7.5 hours earlier than rule-based routine screening protocols [53].

### Treatment Period

#### Earlier Treatment and Prioritization of High-Risk Patients

Earlier treatment facilitated by MLAs was reported in 10 articles [28,31,33,36,37,40,49,52,53,57], including 7 quantitative studies (4 prospective [28,31,49,52] and 3 retrospective [33,40,53]), 1 mixed methods study [36], 1 qualitative study [37], and 1 institutional news report [57]. One retrospective study reported that the used MLA enabled earlier treatment up to 40 hours before the onset of sepsis [40], while another indicated that the use of ML may reduce the time to treatment, not providing a specific time reduction [33]. Earlier treatment was reported to enable intervention before or during clinical deterioration [53] and potentially prevent sepsis progression [52]. It may allow for early identification and intervention of patients at high risk for severe sepsis prior to clinical onset [31]. Additionally, the literature highlighted early identification and control of the pathogen causing sepsis [52]. Detecting patients before the onset of septic shock may facilitate earlier clinical assessment, diagnostic testing, therapeutic interventions, and transfer to appropriate levels of care [53]. Ultimately, earlier treatment may improve patient outcomes [37] and lead to an alteration in the prevalence of septic shock through timely intervention by health care providers from 5.3% in the control group to 1.5% in the experimental group (–71.7%) of the corresponding study [49]. MLAs were also associated with shorter times to obtain blood cultures (0.98‐2.79 hours) [49,57], fluid administration (1.05 hours) [57] and earlier administration or adjustment of antibiotics (0.55‐2.76) [28,36,49,57]. A positive correlation between timely evaluation of MLA alerts and quicker administration of antibiotics was reported, as earlier evaluation of alerts leads to faster use of antibiotics [36]. Finally, a quantitative prospective study reported that MLAs allow prioritization of high-risk patients, with the targeted real-time early warning system (TREWS) identifying who is most likely to benefit from timely treatment [28].

#### Individualized Therapy

MLAs were reported to support individualized, patient-centered therapy in 11 articles [29,30,33,39,41,44-47,50,54], comprising 10 quantitative studies (1 prospective [54] and 9 retrospective [29,30,33,39,41,45-47,50]) and 1 review [44]. Four main approaches for individualization were identified: (1) subgroup analyses and clustering, (2) optimized substance administration, (3) personalized nursing care, and (4) general statements. Three articles reported subgroup analyses and clustering of patients [41,45,46], which may enable hospitals to provide targeted treatments tailored to the specific needs of defined subgroups [45] and classify patients according to their diverging mortality risk due to factors such as fluid overload or norepinephrine overdose. Such classification might support the development of tailored resuscitation strategies for patients with septic shock [41]. Furthermore, subgroup analyses applied to populations with differing disease severity and progression allow MLAs to adjust the intensity of therapy [46]. The use of MLAs for optimal substance administration was reported in 6 articles [29,33,39,41,50,54]. Applications include personalized antibiotic dosing [33], faster adjustment to the most effective antibiotics, and drug resistance prediction. The comprehensive Intelligent Antimicrobial System demonstrated potentially faster drug resistance prediction times compared with conventional methods, requiring 39.8 hours for carbapenem-resistant Klebsiella pneumonia and 40.9 hours for methicillin-resistant Staphylococcus aureus, compared with 99.5 and 106.4 hours, respectively, using traditional methods [54]. MLA use was also associated with an 8% reduction in antibiotic resistance [50], may shorten the time to antimicrobial resistance detection by 37 hours [54], and was reported to reduce the duration of antibiotic treatment [50]. Furthermore, MLAs may support physicians in selecting appropriate antibiotic therapy [54], with AI-based antibiotic stewardship linked to decreased *Clostridium difficile* infections [50]. Beyond antibiotics, MLAs have demonstrated utility in optimizing dosing strategies for norepinephrine [41], vasopressors [39], corticosteroids [29], and fluid volume management [41]. MLAs also reported to enhance nursing competence and support more evidence-based, personalized nursing care [47]. General statements on individualized therapy were identified in 4 articles [30,33,44,54], including personalized treatment to support physicians in diagnosing and managing bacteremia [33], facilitation of shared decision-making through preoperative discussions [30], improved physician adherence [44], and more precise treatment tailored to individual patients [54].

#### Improved Patient Outcomes

##### Improved SOFA Score

Improved SOFA scores associated with the application of and timely response to MLAs were reported in 1 quantitative prospective study [28]. The SOFA score, the predominant measure for assessing the severity of organ dysfunction, is closely linked to the probability of mortality, with a higher score indicating an increased probability of death [3,58]. Using the TREWS algorithm, Adams et al [28] reported a SOFA score progression of –0.8 in their intervention group, compared to –0.4 in the control group. The article highlights a disproportionate reduction in the SOFA score for high-risk patients compared to nonhigh-risk patients. Additionally, timely evaluation and confirmation of the TREWS alerts is associated with improvements in SOFA score progression.

##### Reduced Length of Stay

Seven quantitative articles reported reductions in LOS [28,31,43,49,50,52,54], including 6 prospective [28,31,43,49,52,54] and 1 retrospective study [50]. Based on the identified literature, a distinction can be drawn between (1) specific reductions, reported in absolute or relative terms [28,31,43,49,52,54], and (2) general statements without precise quantification [28,49,50]. Reported specific reductions in hospital LOS ranged from 0.43 to 8.1 days [28,31,43,49,52], corresponding to decreases of 12.84%-45.25% [31,43,49,52]. Reported reductions in ICU LOS varied between 2.09 and 10.5 days [49,50]. One study also highlighted that shorter ICU stays may contribute to an overall reduction in hospital LOS, although LOS on the general ward increased by 2.4 days [50]. Another article reported a potential annual reduction of 1100 days in emergency department stays and the prevention of 34 ICU stays associated with MLA usage in the examined hospital [54]. General statements indicated a disproportionate, though not statistically significant, reduction in LOS among high-risk patients as well as reduced LOS when MLA-generated alarms were evaluated and confirmed timely [28]. AI-based antibiotic stewardship was also associated with shorter LOS [50] and MLAs were reported to significantly shorten hospital LOS compared to rule-based systems [49]. Furthermore, 1 study suggested that timely physician responses to MLA-generated alerts may contribute to reduced LOS [28].

##### Reduced Mortality

A reduction in mortality was reported in 14 articles [28,29,31-33,39,43,48,49,52,54-57], including 9 quantitative studies (6 prospective [28,31,43,49,52,54] and 3 retrospective [29,33,39]) as well as 3 reviews [32,48,55] and 2 institutional news reports [56,57]. Reported mortality reductions varied in type and presentation, encompassing (1) specific quantitative statements, expressed in relative or absolute terms [28,29,31,32,43,49,52,54,56,57], and (2) general statements without numerical specifications [28,29,33,39,48,49,54,55]. Relative reductions of mortality ranged from 13.19% to 74.94% [28,31,43,49,52,56,57], whereas absolute reductions ranged from 1.33% points to 26.4% points [28,29,31,43,49,52,54]. One study reported an increase in absolute survival rate of 11.7% and 23.7%, depending on the type of bacteria responsible for the sepsis [54]. Two articles provided reductions in natural numbers; one projected 22 potentially preventable annual deaths in the emergency department of the China Medical University Hospital [54], while another estimated several thousand preventable deaths in the United States alone [32]. General statements suggested that MLAs may disproportionately reduce mortality among high-risk patient cohorts, particularly when outputs are promptly evaluated and confirmed by physicians [28]. Improved survival rates may also be linked to the use of MLA-guided antibiotic recommendations [54] and the application of the 3PM (predictive, preventive, and personalized medicine) principles [33]. MLAs are associated with lower mortality compared to traditional physician assessments [29,39] and predictions generated by rule-based tools [49]. Additionally, literature provided general statements, offering limited informational depth and indicating that the use of ML may contribute to reduced mortality [29,39,48,49,55].

### General Improvements in Care

Eight articles reported improvements in care associated with MLAs [31,34,38,43,47-49,54], including 6 quantitative studies (5 prospective [31,34,43,49,54] and 1 retrospective [47]) as well as 2 reviews [38,48]. Reported benefits can be divided into 2 domains: (1) statements related to time and (2) statements on patient care enhancements. One study reported a reduced duration of septic shock [48]. Within the patient care enhancement category, MLAs were described as posing no risk to patients and offering potential benefits to patients and health care providers [31], reducing events of clinical deterioration [38], improving care accuracy [47,54], and increasing sepsis awareness among physicians [43,49]. Physicians and nurses also reported perceived improvements in care [34].

### Posttreatment Period (Reduced Readmission Rate)

Predictive AI-based CDSS were associated with reduced 30-day readmission rates, as reported in 2 quantitative prospective studies [31,43]. In Burdick et al [31], implementation of an MLA reduced the 30-day readmissions from 36.4% to 28.12%, representing a 22.74% reduction compared to the baseline period. McCoy and Das [43] reported a decline from 46.19% (188/407) during the preimplementation baseline period to 29.8% (100/336) in a first postimplementation period and further to 25.2% (96/381) in a second postimplementation period. In a subsequent steady-state period, the 30-day readmission rate was further reduced to 7.84% (16/204). Across all surveyed months after implementation, the 30-day readmission rate was 23.03%, representing a 50.14% reduction in the sepsis-related 30-day readmission rate.

## Discussion

### Principal Findings

This scoping review presents the evidence on the patient-relevant benefits of AI-based CDSS in sepsis care. All articles focusing on sepsis and presenting the influence of AI-based CDSS on patient-relevant benefits, identified through the comprehensive search strategy, were included. In total, 30 articles were identified and integrated into the review. Investigating the literature, there is a number of AI-based CDSS for sepsis treatment developed in the past or currently under development. However, research typically has no or only limited reference to patient-relevant benefits and (1) mostly focuses on problems and/or success strategies [32,55,59-61] and/or (2) is indication-independent [59]. To the best of the authors’ knowledge, this represents the first scoping review on this specific topic.

The findings of this scoping review, systematized into the 6 main categories, (1) prediction, (2) earlier treatment and prioritization of high-risk patients, (3) individualized therapy (which encompasses patient-centered care), (4) improved patient outcomes (which includes improved SOFA score, reduced length of stay, and reduced mortality), (5) general improvements in care, and (6) reduced readmission rate, underscore the potential patient-relevant benefits of AI-based CDSS in sepsis care across the entire inpatient pathway. The literature indicates that MLAs can potentially predict sepsis before its clinical onset [35,38,40,51,56]. Additionally, septic shock [42,51] and sepsis-related organ dysfunction [53] may be predicted in advance. These predictive capabilities can contribute to reducing the incidence of septic shock [38] and supporting decreased mortality rates among sepsis patients [12]. Sepsis prediction may facilitate timely treatment initiation through the use of MLAs [40]. This was associated with improved patient outcomes and a decreased prevalence of septic shock [49]. Furthermore, individualized therapy can potentially have a positive impact on patient-relevant benefits by reducing the time to treatment or LOS for each individual patient [33]. Moreover, the disproportionate reduction in the SOFA score through the use of ML compared to a control group whose treatment was not supported by MLAs should be mentioned. According to the Sepsis-3 definition, the level of the SOFA score positively correlates with the probability of death [3], and a SOFA score of ≥2 points corresponds to a mortality risk of over 10% in hospitalized patients outside the ICU [25]. The TREWS algorithm presented by Adams et al was able to reduce the SOFA score by 0.8 points, while a reduction of only 0.4 points was observed in the control group. Accordingly, the use of this MLA may contribute to the reduction in mortality. In general, the usage of MLAs was associated with a mortality reduction of up to 74.94% [28,31,43,49,52,56,57], with faster response times being associated with greater reductions in mortality [28]. This demonstrates the medical potential of ML in the treatment of sepsis, particularly when clinical recommendations are accepted and promptly implemented by physicians. With approximately 11 million deaths annually from sepsis according to the WHO [2], a corresponding reduction in mortality could translate into a substantial global health impact. Additionally, MLAs were linked to reduced hospital LOS [31,43,49,52] and ICU LOS [49,50]. Beyond their predictive capabilities, facilitation of timely treatment, mortality, and LOS reductions, AI-based CDSS in sepsis care provide further patient benefits, including shortened duration of septic shock [48], reduced antibiotic resistance, and reduced duration of antibiotic treatment [50]. MLAs may also contribute to a reduction of events of clinical deterioration [38] and increased physician awareness of sepsis [43,49]. Finally, the literature indicates that AI-based CDSS in sepsis care can contribute to reducing hospital readmission rates [43], further demonstrating their potential to improve patient-relevant benefits.

### Comparison With Prior Work

While 6 reviews were included in this work, they primarily focused on other topics and predominantly used less systematic approaches [32,38,44,48,51,55]. Among the included reviews, 4 adopted a narrative review methodology [32,44,48,51]. By design, this approach is inherently less systematic than systematic reviews or scoping reviews, and this was evident in the search and selection process of the included narrative reviews. Two relied exclusively on limited, nonsystematic keyword searches, one using 8 keywords across 4 search engines [44] and another restricted to 3 keywords in a single database [32]. Moreover, the review conducted by Ferreira et al [32] focused primarily on problems and success strategies related to AI-based CDSS, thereby addressing a different thematic focus than the present scoping review. Another narrative review applied a brief and partial search string without predefined inclusion and exclusion criteria and was limited to a single database [51], representing considerable methodological limitations relative to the present comprehensive scoping review. The narrative review conducted by Schinkel et al [48] adopted a more systematic approach, using a predefined search string and assessing the clinical value of AI-based systems by evaluating the AUROC as a criterion for article selection. While methodologically more robust, this review nonetheless differed from the present article, as it primarily evaluated the advantage of MLAs over rule-based scores, reflecting the status quo using a binary classifier. The advantage of MLAs over rule-based scores was considered a prerequisite for AI-based CDSS in the present study. With the exception of 1 review, where a manual search of reference lists was conducted [32], none of the narrative reviews [44,48,51] undertook a comprehensive search for gray literature or an analysis of the reference lists. Furthermore, only 1 narrative review reported a screening process conducted by 2 independent reviewers [48], whereas the other 3 reviews did not provide methodological detail [32,44,51]. By contrast, the present scoping review implemented a rigorous screening process with 2 independent reviewers to enhance objectivity, reliability, and reproducibility. Beyond these narrative reviews, 1 study followed an integrative review approach, explicitly focusing on predictive algorithms and embedding this narrow focus within a brief predefined search string [38]. In contrast, the present exploratory scoping review aimed to inductively derive patient benefit categories associated with AI-based CDSS in sepsis care. This integrative review relied on a single reviewer for screening [38], representing a methodological limitation in comparison with the dual-reviewer approach of the present scoping review. Finally, 1 systematic review included in this study used a largely rigorous and systematic methodology, with the notable exception of a gray literature search, which was not reported. In addition, this systematic review focused primarily on problems and success strategies [55], thereby diverging from the present scoping reviews’ explicit focus on patient-relevant benefits. In sum, the present scoping review can be clearly distinguished from the included reviews both methodologically and thematically. By applying a comprehensive, exploratory design, centered on patient-relevant benefits, it makes a substantive and valuable contribution to closing the research gap regarding patient-relevant benefits of AI-based CDSS in sepsis care.

### Implications and Recommendations

Patient-relevant benefits identified in the literature are not sufficient to ensure successful implementation of AI-based CDSS. Equally critical is the acceptance of the underlying technology by health care providers and their belief that its use possesses tangible benefits. The unified theory of acceptance and use of technology (UTAUT) provides a framework to understand factors influencing behavioral intention and use behavior using four constructs: (1) performance expectancy, (2) effort expectancy, (3) social influence, and (4) facilitating conditions. In this context, effective design of AI-based CDSS should ensure that providers perceive the system as both beneficial and easy to use, corresponding to the first 2 constructs of the UTAUT. Specifically, (1) users should believe that using AI-based CDSS enhances gains in job performance, and (2) the system is intuitive and easy to operate. Equally important are contextual factors: health care providers should perceive that (3) important others endorse system use, and (4) organizational and technical infrastructure is in place to support usage [61]. A meta-analysis by Dingel et al [62] applying the UTAUT to health care practitioners’ intention to use AI-enabled CDSS confirms that implementation must address not only technical and organizational aspects but also psychological and social factors, particularly fostering user trust. Successful implementation of AI-based CDSS therefore depends only partly on system performance; it is largely contingent on user attitudes and framework conditions.

Beyond the 4 UTAUT constructs, specific barriers [63] and facilitators [64] must be considered when evaluating AI-based CDSS. A nuanced understanding of these factors is essential to accurately evaluate the potential impact of AI-based CDSS on sepsis care. The current evidence demonstrates a pronounced lack of prospective studies investigating the optimal integration of such systems [29]. This paucity of implementation-oriented research, coupled with limited clinician acceptance [37] and insufficient knowledge of AI among health care providers [65], constitutes a substantial barrier to clinical adoption. Concurrently, extant literature highlights pivotal facilitators, emphasizing the importance of prioritizing research on effective integration strategies [38]. For instance, low acceptance may be mitigated by involving health care providers directly in the design and development of CDSS [66-68], while targeted training and educational programs could address knowledge gaps among service providers and enhance trust in this technology [37,67]. These factors must therefore be carefully considered by all stakeholders involved in implementation (eg, caregivers, physicians, and researchers) before real-world adoption can occur. For clinicians, the findings provide insights into realistic benefits, current limitations, and evidence gaps that may guide expectations in clinical decision-making. For researchers, this review underscores the importance of conducting prospective studies and fostering user-centered development to ensure that CDSS effectively translate into clinical practice. In addition, although patient-relevant benefits—and not only measurable patient-relevant outcomes—have been investigated, the findings may contribute to the development of a consistent set of generic patient-relevant outcomes, as proposed by Kersting et al [69]. This could, in turn, facilitate a shared understanding and enhance comparability across studies targeting patient-relevant outcomes, particularly given the absence of a clear, widely accepted definition and standardized criteria for selecting such outcomes.

### Strengths and Limitations

This scoping review was conducted by an interdisciplinary team comprising computer scientists, physicians, statisticians, and (health) economists. This diverse expertise facilitated a comprehensive examination of all relevant aspects across these fields, ensuring a thorough evaluation of the reviewed literature. To address the interdisciplinary research question comprehensively, 4 databases focusing on medicine and informatics were included in the review. In addition, the structured search for gray literature targeted various institutions in 8 countries, including each country’s Ministry of Health, diverse sepsis and intensive care associations for each country, and diverse health informatics associations for each country. The 8 countries were selected based on two criteria: (1) having highly digitalized health care systems and/or (2) holding the status of industrialized nations. These countries were presumed to have a higher likelihood of using AI-based systems. Furthermore, the search encompassed internationally active stakeholders, such as the WHO and OECD, alongside globally active health informatics organizations and sepsis and critical care associations.

Despite all efforts, this scoping review is not free of limitations. Given the exploratory nature of the methodology, publication bias must be considered a potential limitation [70]. Studies in which AI-based CDSS do not demonstrate improvements in patient-relevant benefits compared with conventional scores may not be submitted in peer-reviewed journals, potentially leading to an overestimation of their true patient benefit. Although no formal risk of bias assessment was conducted, the included studies demonstrated considerable heterogeneity in design. Moreover, 65% (13/20) of the quantitative studies relied solely on retrospective methodologies, in which evidence of patient benefits was demonstrated only theoretically. Consequently, the findings of this scoping review should be interpreted with caution, as the reported effects may be overestimated in the context of real-world care. The overall strength of evidence was limited by the predominance of retrospective study designs and the theoretical nature of reported benefits. In contrast, the included prospective studies provided more robust support for the identified benefit categories. Importantly, each benefit category has been substantiated in prospective studies, thereby affirming its validity in real-world clinical contexts rather than solely theoretically in retrospective or descriptive studies (Multimedia Appendix 9). Detailed information on the study designs of all included articles is provided in Multimedia Appendix 6. Multimedia Appendix 9 summarizes benefit categories identified across the respective study designs. Furthermore, the comparability of the reported MLA performance across articles is limited due to varying definitions of sepsis (eg, different causative pathogens, divergent sepsis definitions, and variations in the examined indications such as sepsis, septic shock, or sepsis-related organ dysfunction). The same limitation applies to the databases used for training and validation, which differed substantially in size. In addition, no assessment of the applied MLA methods was conducted, nor was the level of maturity of the individual MLAs explicitly considered. Furthermore, due to the heterogeneity of the included studies, no formal quality assessment was conducted. Rather, the present review was designed to exploratively map and comparatively present the entirety of available evidence in order to identify research gaps, without imposing methodological restrictions on the literature to be included [20-22]. Finally, a methodological limitation should be noted: Research conducted on institutional websites could only be partially conducted for Estonia, Denmark, and Spain due to language restrictions (English and German), as some stakeholder websites were available exclusively in the respective national languages. The utilization of translation tools was deliberately avoided, as the inclusion of material that none of the authors could fully comprehend and critically appraise in the original language was considered methodologically inappropriate.

The search restriction of 15 years should not be considered a limitation. The inclusion period was defined in consultation with ML experts (NT, HN), and algorithms developed prior to the review period (January 1, 2008-March 2, 2023) were predominantly anticipated to be (1) rule-based systems and/or nonblack-box systems for the users. Both types of algorithms are outside the scope of this review. Moreover, an initial limited search in the databases MEDLINE via PubMed and Embase, which accounted for approximately 75% of the screened literature (Figure 1), indicated that only a marginal proportion of articles relevant to the research question were published before 2008. Consequently, the time restriction is unlikely to have affected the identification of relevant literature.

### Conclusion

The findings of this scoping review highlight the considerable medical relevance of AI-based CDSS in sepsis care. These systems offer benefits across the entire patient care pathway, from early detection and risk stratification to individualized therapy and various improved outcomes. AI-based CDSS has shown the ability to predict sepsis, septic shock, and sepsis-related organ dysfunction, enabling earlier initiation of treatment, prioritization of high-risk patients, and tailored therapeutic strategies. In addition to supporting earlier and more targeted interventions, AI-based CDSS contribute to better clinical outcomes, including improved SOFA scores, reduced LOS both in general wards and ICUs, and lower mortality rates. They may also help reduce readmission rates among sepsis patients, further enhancing long-term care quality. With their transformative potential, AI-based CDSS could fundamentally improve the global management of sepsis. However, further research is needed to optimize the development, implementation, and clinical application of these systems to maximize patient benefits and further improve outcomes for sepsis patients in the future. This is particularly important given the highly heterogeneous evidence base, with a substantial proportion of studies relying on retrospective data, as the results of the included studies cannot be directly generalized or applied without caution.

## Supplementary material

10.2196/76772 Multimedia Appendix 1 Search strategy – Medline via PubMed.

10.2196/76772 Multimedia Appendix 2 Search strategy – Embase.

10.2196/76772 Multimedia Appendix 3 Search strategy – ACM Digital Library.

10.2196/76772 Multimedia Appendix 4 Search strategy – IEEE Xplore.

10.2196/76772 Multimedia Appendix 5 Included websites in structured search.

10.2196/76772 Multimedia Appendix 6 Overview of included articles.

10.2196/76772 Multimedia Appendix 7 Patient benefits mentioned in articles.

10.2196/76772 Multimedia Appendix 8 Screened articles.

10.2196/76772 Multimedia Appendix 9 Used methods per benefit category.

10.2196/76772 Checklist 1 PRISMA-ScR checklist.

## Abbreviations

3PM: predictive, preventive, and personalized medicine
AI: artificial intelligence
APACHE II: Acute Physiology and Chronic Health Evaluation II
CDSS: clinical decision support system
ICU: intensive care unit
LOS: length of stay
ML: machine learning
MLA: machine learning algorithm
NEWS2: National Early Warning Score 2
OECD: Organisation for Economic Co-operation and Development
PCC: Population, Concept, Context
PRISMA-ScR: Preferred Reporting Items for Systematic Reviews and Meta-Analyses extension for Scoping Reviews
qSOFA score: Quick Sequential Organ Failure Assessment Score
SIRS: systemic inflammatory response syndrome
SOFA score: Sequential Organ Failure Assessment Score
TREWS: targeted real-time early warning system
UTAUT: unified theory of acceptance and use of technology
WHO: World Health Organization
