# Supplementary material for: Patient Benefits in the Context of Sepsis-Related AI-Based Clinical Decision Support Systems: Scoping Review
Source: J Med Internet Res. 2026 Jan 26;28:e76772. doi: 10.2196/76772 (PMC12834200; doi:10.2196/76772)
Supplement: Multimedia Appendix 1 [file jmir-v28-e76772-s001.docx]

## Multimedia Appendix 2. Search Strategy – Medline via PubMed.

| **Database** | Medline |
| --- | --- |
| **Platform** | PubMed |
| **Date of search** | 02 March, 2023 |
| **Filter** | No filters |

(

systemic inflammatory response syndrome [majr] OR

systemic inflammatory response syndrome* [tiab] OR

sirs [tiab] OR

sepsis [tiab] OR

septicaemi* [tiab] OR

septicemi* [tiab] OR

bloodstream infection* [tiab] OR

blood infection* [tiab] OR

bloodstream poison* [tiab] OR

blood poison* [tiab] OR

sequential organ failure assessment score* [tiab] OR

sofa [tiab] OR

qsofa [tiab] OR

quicksofa [tiab]

)

**AND**

(

decision support systems, clinical [majr] OR

decision making, computer-assisted [majr] OR

decision support techniques [majr] OR

medical informatics computing [majr] OR

medical informatics computing [tiab] OR

diagnosis, computer-assisted [majr] OR

cdss [tiab] OR

cds-system* [tiab] OR

eds-tool* [tiab] OR

support system* [tiab] OR

detection* [tiab] OR

diagnosis [majr] OR

diagnos* [tiab] OR

therapeutics [majr] OR

therap* [tiab] OR

decision* [tiab] OR

predict* [tiab] OR

prognosis [majr] OR

prognos* [tiab] OR

information retrieval* [tiab]

)

**AND**

(

artificial intelligence [majr] OR

artificial intelligence [tiab] OR

machine intelligence [tiab] OR

computational intelligence [tiab] OR

ai [tiab] OR

((deep [tiab] OR machine [tiab] OR unsupervis* [tiab] OR supervis* [tiab] OR reinforc* [tiab]) AND learning [tiab]) OR

neural network* [tiab] OR

natural language processing [tiab] OR

nlp [tiab] OR

medical language processing [tiab] OR

mlp [tiab] OR

text mining [tiab] OR

pattern recognition, automated [majr] OR

automatic pattern recognition* [tiab] OR

automated pattern recognition* [tiab] OR

image processing, computer-assisted [majr] OR

(image [tiab] AND (recognition [tiab] OR classification [tiab] OR processing [tiab])) OR

((machine [tiab] OR computer [tiab]) AND vision [tiab]) OR

data mining [majr] OR

data mining [tiab] OR

data science [majr] OR

data science [tiab] OR

data driven [tiab]

)
