# Supplementary material for: Patient Benefits in the Context of Sepsis-Related AI-Based Clinical Decision Support Systems: Scoping Review
Source: J Med Internet Res. 2026 Jan 26;28:e76772. doi: 10.2196/76772 (PMC12834200; doi:10.2196/76772)
Supplement: Multimedia Appendix 2 [file jmir-v28-e76772-s002.docx]

## Multimedia Appendix 3. Search Strategy – Embase.

| **Database** | Embase |
| --- | --- |
| **Platform** | Embase |
| **Date of search** | 02 March, 2023 |
| **Filter** | No filters |

(

'systemic inflammatory response syndrome'/exp/mj OR

'systemic inflammatory response syndrome*':ti,ab OR

'sirs':ti,ab OR

'sepsis':ti,ab OR

'septicaemia*':ti,ab OR

'septicemia*':ti,ab OR

'bloodstream infection'/exp/mj OR

'blood* infection*':ti,ab OR

'blood* poison*':ti,ab OR

'sequential organ failure assessment score'/exp/mj OR

'sequential organ failure assessment score*':ti,ab OR

'sofa':ti,ab OR

'qsofa':ti,ab OR

'quicksofa':ti,ab

)

**AND**

(

'decision support system'/exp/mj OR

'medical informatics'/exp/mj OR

'medical informatics':ti,ab OR

'computer assisted diagnosis'/exp/mj OR

'cdss':ti,ab OR

'cds system*':ti,ab OR

'eds tool*':ti,ab OR

'support system*':ti,ab OR

'detection*':ti,ab OR

'diagnosis'/exp/mj OR

'diagnos*':ti,ab OR

'therapy'/exp/mj OR

'therap*':ti,ab OR

'decision*':ti,ab OR

'computer prediction'/exp/mj OR

'prediction'/exp/mj OR

'predict*':ti,ab OR

'prognosis'/exp/mj OR

'prognos*':ti,ab OR

'information retrieval'/exp/mj OR

'information retrieval*':ti,ab

)

**AND**

(

'artificial intelligence'/exp/mj OR

'artificial intelligence':ti,ab OR

'machine intelligence':ti,ab OR

'comput* intelligence':ti,ab OR

'ai':ti,ab OR

'machine learning'/exp/mj OR

(('deep':ti,ab OR 'machine':ti,ab OR 'unsupervis*':ti,ab OR 'supervis*':ti,ab OR 'reinforc*':ti,ab) AND 'learning':ti,ab) OR

'neural network*':ti,ab OR

'natural language processing'/exp/mj OR

'natural language processing':ti,ab OR

'nlp':ti,ab OR

'medical language processing':ti,ab OR

'mlp':ti,ab OR

'text mining':ti,ab OR

'automat* pattern recognition*':ti,ab OR

'image processing'/exp/mj OR

('image':ti,ab AND ('recognition':ti,ab OR 'classification':ti,ab OR 'processing':ti,ab)) OR

'computer vision'/exp/mj OR

(('machine':ti,ab OR 'computer':ti,ab) AND 'vision':ti,ab) OR

'data mining':ti,ab OR

'data science'/exp/mj OR

'data science':ti,ab OR

'data driven':ti,ab

)
