# Supplementary material for: Patient Benefits in the Context of Sepsis-Related AI-Based Clinical Decision Support Systems: Scoping Review
Source: J Med Internet Res. 2026 Jan 26;28:e76772. doi: 10.2196/76772 (PMC12834200; doi:10.2196/76772)
Supplement: Multimedia Appendix 3 [file jmir-v28-e76772-s003.docx]

## Multimedia Appendix 4. Search Strategy – ACM Digital Library.

| **Database** | The ACM Guide to Computing Literature |
| --- | --- |
| **Platform** | ACM Digital Library |
| **Date of search** | 02 March, 2023 |
| **Filter** | No filters |

(

"systemic inflammatory response syndrome" OR

"systemic inflammatory response syndromes" OR

sirs OR

sepsis OR

septicaemi* OR

septicemi* OR

"bloodstream infection" OR

"bloodstream infections" OR

"blood infection" OR

"blood infections" OR

"bloodstream poison" OR

"bloodstream poisons" OR

"bloodstream poisoning" OR

"blood poison" OR

"blood poisons" OR

"blood poisoning" OR

"sequential organ failure assessment score" OR

"sequential organ failure assessment scores" OR

sofa OR

qsofa OR

quicksofa

)

**AND**

(

"medical informatics computing" OR

cdss OR

"cds-system" OR

"cds-systems" OR

"eds-tool" OR

"eds-tools" OR

"support system" OR

"support systems" OR

detection* OR

diagnos* OR

therap* OR

decision* OR

predict* OR

prognos* OR

"information retrieval"

)

**AND**

(

"artificial intelligence" OR

"machine intelligence" OR

"computational intelligence" OR

ai OR

((deep OR machine OR unsupervis* OR supervis* OR reinforc*) AND learning) OR

"neural network" OR

"neural networks" OR

"natural language processing" OR

nlp OR

"medical language processing" OR

mlp OR

"text-mining" OR

"automatic pattern recognition" OR

"automated pattern recognition" OR

(image AND (recognition OR classification OR processing)) OR

((machine OR computer) AND vision) OR

"data-mining" OR

"data-science" OR

"data-driven"

)
