# Supplementary material for: Patient Benefits in the Context of Sepsis-Related AI-Based Clinical Decision Support Systems: Scoping Review
Source: J Med Internet Res. 2026 Jan 26;28:e76772. doi: 10.2196/76772 (PMC12834200; doi:10.2196/76772)
Supplement: Multimedia Appendix 4 [file jmir-v28-e76772-s004.docx]

## Multimedia Appendix 5. Search Strategy – IEEE Xplore.

| **Database** | IEEE Xplore |
| --- | --- |
| **Platform** | IEEE Xplore |
| **Date of search** | 02 March, 2023 |
| **Filter** | No filters |

(

"systemic inflammatory response syndrome" OR

"systemic inflammatory response syndromes" OR

sirs OR

sepsis OR

septicaemia OR

septicaemias OR

septicemia OR

septicemias OR

"bloodstream infection" OR

"bloodstream infections" OR

"blood infection" OR

"blood infections" OR

"bloodstream poison" OR

"bloodstream poisons" OR

"bloodstream poisoning" OR

"blood poison" OR

"blood poisons" OR

"blood poisoning" OR

"sequential organ failure assessment score" OR

"sequential organ failure assessment scores" OR

sofa OR

qsofa OR

quicksofa

)

**AND**

(

"medical informatics computing" OR

cdss OR

"cds system" OR

"cds systems" OR

"eds tool" OR

"eds tools" OR

"support system*" OR

detection OR

detections OR

diagnos* OR

therapy OR

therapies OR

decision OR

decisions OR

predict* OR

prognosis OR

prognoses OR

"information retrieval"

)

AND

(

"artificial intelligence" OR

"machine intelligence" OR

"comput* intelligence" OR

ai OR

((deep OR machine OR unsupervis* OR supervis* OR reinforc*) AND learning) OR

"neural network*" OR

"natural language processing" OR

nlp OR

"medical language processing" OR

mlp OR

"text mining" OR

"automatic pattern recognition" OR

"automated pattern recognition" OR

(image AND (recognition OR classification OR processing)) OR

((machine OR computer) AND vision) OR

"data mining" OR

"data science" OR

"data driven"

)

**Note on peculiarities of the syntax:**

A total of 8 wildcards are available in the IEEE Xplore database. Following an initial search, these were assigned to the search terms that appeared most relevant for the scoping review:

- support system*
- comput* intelligence
- diagnos*
- predict*
- unsupervis*
- supervis*
- reinforc*
- neural network*
