# Supplementary material for: Patient Benefits in the Context of Sepsis-Related AI-Based Clinical Decision Support Systems: Scoping Review
Source: J Med Internet Res. 2026 Jan 26;28:e76772. doi: 10.2196/76772 (PMC12834200; doi:10.2196/76772)
Supplement: Multimedia Appendix 5 [file jmir-v28-e76772-s005.docx]

## Multimedia Appendix 6. Included websites in Structured Search.

**Researched institutions for each country**

- Ministries of Health at national level
- National sepsis associations
- National associations for intensive care medicine
- National medical informatics associations

**Search terms for gray literature search**

| **German (for German stakeholders)** | **English** |
| --- | --- |
| Sepsis | sepsis |
| septisch | septic |
| Künstliche Intelligenz | artificial intelligence |
| Maschinelles Lernen | machine learning |
| Maschinellem Lernen |  |
| Maschinellen Lernen |  |
| Maschinenlernen |  |
| CDSS | CDSS |
| Entscheidungsunterstützung | Decision Support |

**Note on the selection of countries**

The selection of countries included in the search was based on the results of the Bertelsmann #SmartHealthSystem study which examined the degree of digitalization of various health care systems in 2018. It was assumed that the prospect of identifying information about AI-based CDSS would be particularly high in countries with highly digitalized health care systems. Even though the study states that Estonia has the most digitalized health care system of the countries surveyed, Estonia was only partially included in the gray literature research, as, due to language restrictions, no national stakeholders were identifiable apart from the Ministry of Health, whose website offered no information on the research questions.

**Overview of searched websites**

| Germany | Ministry of Health | Bundesministerium für Gesundheit | <https://www.bundesgesundheitsministerium.de/index.html> |
| --- | --- | --- | --- |
|  | National sepsis associations and associations for intensive care medicine | Deutsche Sepsis Gesellschaft | <https://www.sepsis-gesellschaft.de/> |
|  |  | Sepsis Stiftung | <https://sepsis-stiftung.de/> |
|  |  | Deutsche Interdisziplinäre Vereinigung für Intensiv- und Notfallmedizin | <https://www.divi.de/> |
|  |  | Deutsche Gesellschaft für Anästhesiologie und Intensivmedizin | <https://www.dgai.de/> |
|  |  | Deutsche Gesellschaft für Internistische Intensivmedizin und Notfallmedizin | <https://www.dgiin.de/> |
|  | National medical informatics associations | Medizininformatik-Initiative | <https://www.medizininformatik-initiative.de/de/start> |
|  |  | Deutsche Gesellschaft für Medizinische Informatik, Biometrie und Epidemiologie | <https://www.gmds.de/> |
| USA | Ministry of Health | Food and Drug Administration | <https://www.fda.gov/> |
|  | National sepsis associations and associations for intensive care medicine | Sepsis Alliance | <https://www.sepsis.org/> |
|  |  | Society of Critical Care Medicine | <https://www.sccm.org/Home> |
|  |  | Society for Critical Care Anesthesiologists | <https://socca.org/> |
|  |  | American Association of Critical-Care Nurses | <https://www.aacn.org/> |
|  | National medical informatics associations | American Medical Informatics Association | <https://amia.org/> |
|  |  | Healthcare Information and Management Systems Society | <https://www.himss.org/> |
|  |  | American Health Information Management Association | <https://www.ahima.org/> |
|  |  | American Nursing Informatics Association | <https://www.ania.org/> |
| UK | Ministry of Health | Department of Health and Social Care | <https://www.gov.uk/government/organisations/department-of-health-and-social-care> |
|  | National sepsis associations and associations for intensive care medicine | The UK Sepsis Trust | <https://sepsistrust.org/> |
|  |  | Intensive Care Society | <https://ics.ac.uk/> |
|  |  | Royal College of Anaesthetists | <https://www.rcoa.ac.uk/> |
|  |  | Faculty of Intensive Care Medicine | <https://www.ficm.ac.uk/> |
|  |  | British Association of Critical Care Nurses | <https://www.baccn.org/> |
|  | National medical informatics associations | British Computer Society Health and Care | <https://www.bcs.org/membership-and-registrations/member-communities/bcs-health-and-care> |
|  |  | Faculty of Clinical Informatics | <https://fci.org.uk/> |
|  |  | Digital Health and Care Alliance | <https://dhaca.org.uk/> |
| Denmark | Ministry of Health | Indenrigs- og Sundhedsministeriet | <https://ism.dk/english> |
|  | National sepsis associations and associations for intensive care medicine | Dansk Selskab for Infektionsmedicin | <https://www.infmed.dk/english> |
|  |  | Dansk Selskab for Anæstesiologi og Intensiv Medicin | <https://dasaim.dk/> |
|  | National medical informatics associations | Dansk Selskab for Medicinsk Informatik | Ohne login kann man auf keine Inhalte zugreifen bzw. man wird direkt auf den Login weitergeleitet: <http://www.dsmi.dk/> |
|  |  | Sundhedsdatastyrelsen  (Danish Health Data Authority) | <https://sundhedsdatastyrelsen.dk/da/english> |
| Canada | Ministry of Health | Health Canada | <https://www.canada.ca/en/health-canada.html> |
|  | National sepsis associations and associations for intensive care medicine | Sepsis Canada | <https://digitalhealthcanada.com/about/> |
|  |  | Canadian Sepsis Foundation | <https://www.echima.ca/association/> |
|  |  | Canadian Critical Care Society | <https://www.canadiancriticalcare.org/> |
|  |  | Canadian Association of Critical Care Nurses | <https://caccn.ca/> |
|  | National medical informatics associations | National Institutes of Health Informatics | <https://nihi.ca/> |
|  |  | Canadian Health Informatics Association | ? |
|  |  | Canadian Institute for Health Information | <https://www.cihi.ca/en> |
|  |  | Digital Health Canada | <https://www.sepsiscanada.ca/> |
|  |  | Canadian Health Information Management Association | <https://www.canadiansepsisfoundation.ca/> |
|  |  | Canada Health Infoway | <https://www.infoway-inforoute.ca/en/> |
|  |  | Canadian Nursing Informatics Association | <https://cnia.ca/> |
| Israel | Ministry of Health | Misrad HaBriut | <https://www.gov.il/en/departments/ministry_of_health/govil-landing-page> |
|  | National medical informatics associations | Israeli Association for Medical Informatics | <https://ilami.org/en/> |
| Spain | Ministry of Health | Ministerio de Sanidad | <https://www.sanidad.gob.es/en/home.htm> |
|  | National sepsis associations and associations for intensive care medicine | Sociedad Española de Medicina Intensiva, Crítica y Unidades Coronarias | <https://semicyuc.org/> |
|  |  | Sociedad Española de Anestesiología, Reanimación y Terapéutica del Dolor | <https://www.sedar.es/> |
|  | National medical informatics associations | Sociedad Española de Informática de la Salud | <https://seis.es/> |
| International stakeholders | National sepsis associations and associations for intensive care medicine | Survival Sepsis Campaign | <https://www.sccm.org/SurvivingSepsisCampaign/Home> |
|  |  | Global Sepsis Alliance | <https://globalsepsisalliance.org/> |
|  |  | End Sepsis | <https://www.endsepsis.org/> |
|  |  | International Sepsis Forum | <https://sepsisforum.org/> |
|  |  | World Federation of Societies of Intensive and Critical Care Medicine | <https://www.wficc.com/> |
|  |  | European Society of Intensive Care Medicine | <https://www.esicm.org/> |
|  |  | Society of Critical Care Medicine | <https://www.sccm.org/Home> |
|  | National medical informatics associations | International Medical Informatics Association | <https://imia-medinfo.org/wp/> |
|  |  | European Federation for Medical Informatics | <https://efmi.org> |
|  |  | International Federation of Health Information Management Associations | <https://ifhima.org/> |
|  | World Health Organization | | <https://www.who.int/> |
|  | Organization for Economic Co-operation and Development | | <https://www.oecd.org/> |
