# Supplementary material for: Patient Benefits in the Context of Sepsis-Related AI-Based Clinical Decision Support Systems: Scoping Review
Source: J Med Internet Res. 2026 Jan 26;28:e76772. doi: 10.2196/76772 (PMC12834200; doi:10.2196/76772)
Supplement: Multimedia Appendix 6 [file jmir-v28-e76772-s006.docx]

## Multimedia Appendix 7. Overview of Included Articles.

| **Author(s)** | **Year** | **Country^[[1]](#footnote-1)^** | **Study Population^[[2]](#footnote-2)^ / Validation database** | **Setting^[[3]](#footnote-3)^** | **Training database** | **Intervention** | **Study design** |
| --- | --- | --- | --- | --- | --- | --- | --- |
| **Systematic Research** | | | | | | | |
| Adams et al (28] | 2022 | USA | 590,736 patient encounters.  The study included patients aged ≥18 years who were admitted to the emergency department or hospitalized in 1 of the 5 emergency hospitals between the deployment of TREWS and September 30, 2020.  Each presentation of a patient in the emergency department or on admission was recorded as a single patient encounter. Each encounter was recorded separately. | Two academic and three community hospitals (Howard County General Hospital (HCGH), Suburban Hospital, Bayview Medical Center (BMC), Johns Hopkins Hospital (JHH), Sibley Memorial Hospital). | Retrospective EHR data including 4860 adult patients who were admitted to 3 of the 5 deployment hospitals (HCGH, JHH and BMC) between January 1, 2016, and March 31, 2018, who would have triggered an alert during their stay and who met all inclusion criteria for the primary analysis. | *Targeted Real-time Early Warning System (TREWS)* algorithm based on two separate ridge logistic regression models. | Prospective, multi-center, two-arm cohort study |
| Bologheanu et al [29] | 2023 | Austria | Does not apply. | Does not apply. | Data from the AmsterdamUMCdb database including patients aged ≥18 years with new organ dysfunction (either a SOFA score ≥2 at admission or an increase of 2 points or more in the SOFA score during the ICU stay) were included in the sepsis cohort.  Patients aged <18 years at the time of the ICU admission and patients who stayed in the ICU less than 24 h were excluded. | Reinforcement learning algorithm consisting of two distinct neural networks based on the Markov decision process to optimise the corticosteroid treatment strategy for a given patient condition in critically ill patients with sepsis. | Retrospective observational research database study |
| Bunn et al [30] | 2021 | USA | Does not apply. | Does not apply. | Current Procedural Terminology codes from the American College of Surgeons National Surgical Quality Improvement Program (ACS NSQIP) database, were used to identify cases of open (44950, 44960) and laparoscopic (44970, 44979, 44950, 44960) appendectomy performed as principle operative procedure in patients aged ≥16 years between 2005 and 2017.  96.64% of 223214 NSQIP records included for appendectomy were performed for a primary diagnosis of appendicitis. | Classification algorithms included traditional multivariable logistic regression and 3 machine learning algorithms: support vector machines, random forest decision trees and extreme gradient boosting machines. | Retrospective observational research database study |
| Burdick et al [31] | 2020 | USA | 75147 patient encounters from early 2017 to mid-2018.  Prospectively collected inpatient wards and emergency departments real-world data from patients aged ≥18 years. All genders and ethnicities were included. | 7 teaching and 2 non-teaching hospitals.  **Hospital size:**  n = 3, small (<100 beds); n = 2, medium (100-250 beds); n = 4, large (>250 beds).  **Geographical region:**  n = 1, Northeast; n = 3, South; n = 1, Midwest; n = 4, West. | In 4 of the 9 hospitals, data was collected to measure baseline outcomes before the implementation of the machine learning algorithm (MLA) and for training on the algorithm after its implementation. | Machine learning classifier using gradient boosted trees. | Prospective, multi-center clinical outcomes evaluation |
| Ferreira et al [32] | 2022 | USA | Does not apply. | Does not apply. | Does not apply. | Does not apply. | Narrative Review |
| Garnica et al [33] | 2021 | Spain | Does not apply. | Hospital Universitario de Fuenlabrada, Madrid, Spain (350-bed hospital with the following services: general surgery, urology, orthopaedic surgery, gynaecology and obstetrics, paediatrics, intensive care units (ICUs), haematology-oncology, internal medicine and cardiology). | Database was gathered from 2005 to 2015 and consists of 4357 anonymous patient records containing 117 features per patient, 49.3% female with age 65.1 ± 19.7, and 56.1% male with age 62.7±20.2.  Database contains 2123 bacteraemia (51.3%), which includes aerobic, strict anaerobic and facultative anaerobic bacteria, and 2234 no bacteraemia (48.7%), including 1844 contaminations. | 3 supervised machine learning classifiers: support vector machine, random forest and k-nearest neighbours. | Retrospective observational single-center study |
| Ginestra, et al [34] | 2019 | USA | Non-ICU admissions between November and December 2016 (no further specification).  During the six-week study period, 362 EWS 2.0 alerts were triggered. For the 724 potential first survey responses (one each for a nurse and provider per alert), 287 first surveys were completed by 252 individual clinicians (overall response rate 40%). Nurses completed 180 first surveys (50% response rate) and providers completed 107 first surveys (30% response rate). Of these, 43 nurses who completed a first survey completed a second survey (24% response rate) and 44 providers who completed a first survey completed a second survey (41% response rate), with an overall second survey response rate of 30%. Of these 77 respondents, 49 (64%, 33 providers, 16 nurses) reported sufficient continuity with the alerted patient to accurately complete the second survey. | The MLA was deployed throughout a multi-hospital academic health care system. The study was conducted in the flagship 782-bed tertiary academic teaching hospital in Philadelphia, Pennsylvania, USA. | Electronic health record data from adult patients discharged from July 2011, to June 2014, from three urban acute care hospitals (n=162212).  The MLA was validated on hospitalized patients from October to December 2015 (n=10448, screened positive=314). | The MLA was developed using a random forest classifier trained on electronic health record data.  Nurses and providers were surveyed twice about their perceptions of the MLAs helpfulness and impact on care, first within 6 hours of the alert, and again 48 hours post-alert. | Prospective, single-center observational study |
| Goh et al [35] | 2021 | Singapore | Does not apply. | Does not apply. | MySQL 8.0 was used to extract data from the Epic electronic medical record system. The sample consists of 5317 patients admitted from April 2, 2015, to December 31, 2017, with 114602 clinical note entries.  For a diagnosis algorithm, electronic medical record system clinical notes for each consultation were combined with the most recent structured variables available in the electronic medical record system.  For an early prediction algorithm, the data structure is similar to that of the diagnosis algorithm except that the early prediction algorithm did not consider any patient consultations from the sample when the patients have been confirmed to have sepsis (ie transferred to an ICU).  In addition to structured data used in the predictive model, clinical notes data that is unstructured free-form text was utilized. | The *SERA* MLA consists of two inter-linked algorithms – a diagnosis algorithm (voting ensemble MLA and for comparative purposes dagging and gradient boosted trees) and an early prediction algorithm (voting ensemble MLA).  The diagnosis algorithm determines if the patient has sepsis at the time of consultation and if not, the early prediction algorithm will determine the patient’s risk of having sepsis in the next 4, 6, 12, 24, and 48 h.  Before using unstructured clinical notes as predictors in the MLA unsupervised NLP was apllied using the latent Dirichlet allocation topic modeling algorithm. | Retrospective observational single-center cohort study |
| Henry et al [36] | 2022 | USA | **Study question 1:** 469419 screened patients. All patients presenting to the ED or admitted to an observation or inpatient unit.  **Study questions 2** **and** **3.1:** 3775 patients with sepsis who received an alert and who did not receive an antibiotic order before the alert, but who received antibiotics within 24 hours after the alert.  **Study question 3.2:** 2463 of patients from study questions 2 and 3.1 who had an evaluation of their alert entered by a provider within 3 hours of the alert and who also received antibiotic treatment over the course of 4 days or more.  Included hospitals and date ranges were: Howard County General Hospital (1 April 2018 to 31 March 2020), Suburban Hospital (1 October 2018 to 31 March 2020), Bayview Medical Center (February 1, 2019, to March 31, 2020), Johns Hopkins Hospital (April 1, 2019 to March 31, 2020) and Sibley Memorial Hospital (May 1, 2019 to March 31, 2020). | Multiple hospitals in the Johns Hopkins Health System.  **Retrospective model performance assessment:**  1 academic and 2 community hospitals in Maryland / Washington DC area (Howard County General Hospital, Johns Hopkins Hospital and Bayview Medical Center).  **Prospective analysis of alert response:**  3 community and 2 academic hospitals in the Maryland and DC areas (Howard County General Hospital, Suburban Hospital, Bayview Medical Center, Johns Hopkins Hospital and Sibley Memorial Hospital). | **Database for retrospective cohort:**  173931 patient encounters between January 2016, and March 2018. 3858 sepsis cases were retrospectively identified using EHR-based sepsis phenotyping. | *Targeted Real-time Early Warning System (TREWS)* consisting of a mixture of cox proportional hazard models and several machine learning-based techniques. | Mixed methods approach (retrospective multi-center case control study and prospective multi-center study) |
| Joshi et al [37] | 2022 | USA | 13 semi-structured interviews and a questionnaire were conducted with 21 hospital leaders overseeing clinical decision support implementation at 15 US medical centers. Five of the 13 interviews were conducted simultaneously with more than 1 participant. | 7 community and 8 academic hospitals were included. Adult populations were served in all but 2 hospitals.  **Hospital size:**  n = 3, <300 beds; n = 4, 300-500 beds; n = 8, >500 beds.  **Geographical region:**  n = 9, Northeast; n = 1, Southeast; n = 5, Midwest; n = 1, West coast. | Does not apply. | 7 hospitals employed a ML tool (no further information given) and 8 employed a rule-based tool. Homegrown tools were most common (n = 9), followed by electronic medical record vendor tools (n = 4), and third party tools (n = 2). | Qualitative semi-structured interview study including a questionnaire |
| Kausch et al [38] | 2021 | USA | Does not apply. | Does not apply. | Does not apply. | Does not apply. | Integrative Review |
| Komorowski et al [39] | 2018 | UK | Does not apply. | Does not apply. | Whole MIMIC-III dataset: n = 17083 admissions from 5 separate ICUs in one tertiary teaching hospital.  Test dataset consists of adult patients from eICU Research Institute Database (eRI) fulfilling the sepsis-3 criteria defined as a suspected infection (prescription of antibiotics and sampling of bodily fluids for microbiological culture) combined with evidence of organ dysfunction, defined by a SOFA score ≥2 (n = 79083 admissions from 128 hospitals (n = 37146 nonteaching; n = 29388 teaching; n = 12539 unknown)). | *AI Clinician* based on a reinforcement learning algorithm. | Retrospective observational multi-research database study |
| Kuo et al [40] | 2021 | Taiwan | Does not apply. | Does not apply. | Public domain database consisting of ICU patient records in the Beth Israel Deaconess Medical Center and Emory University Hospital, including a total of 40336 patient records (2932 sepsis and 37404 non-sepsis patients), collected over a ten-year period. | Two-layer feed-forward artificial neural network with sigmoid hidden and softmax output neurons, implemented using the MATLAB artificial neural network toolbox, which was based on stochastic gradient descent. | Retrospective observational research database study |
| Ma et al [41] | 2021 | China | Does not apply. | Does not apply. | 1437 patients from 25 tertiary care teaching hospital in China from January 2016 to December 2017 were included for subsequent analysis.  5856 patients from the eICU Collaborative Research Database (eICU-CRD) were utilized for external model validation. | Non-supervised learning algorithms including finite mixture modelling and k-means clustering to identify different classes of septic shock.  Multivariable cox model to explore interactions between class membership and fluid volume or epinephrine dose. | Retrospective observational multi-center and research database study |
| Mao et al [42] | 2018 | USA | Does not apply. | Does not apply. | **Primary data source:** UCSF, University of California (San Francisco, California, USA).  **Transfer-learning source**:  MIMIC-III v1.3 dataset, compiled from the Beth Israel Deaconess Medical Center (BIDMC) (Boston, Massachusetts, USA).  **Generalizability evaluation:**  Stanford Medical Center (Stanford, California, USA),  Oroville Hospital (Oroville, California, USA),  Bakersfield Heart Hospital (BHH; Bakersfield, California, USA),  Cape Regional Medical Center (CRMC; Cape May Courthouse, New Jersey, USA).  **Inclusion criteria:** Inpatients, ≥18 years with ≥ 1 observation of each required measurement and with a prediction time between 7 and 2000 hours:  UCSF: 90353 patients (June 2011 to March 2016),  MIMIC-III: 21604 patients (2011 to 2012),  Stanford: 239767 patients,  Orovile: 1140 patients,  BHH: 2231 patients,  CRMC: 4295 patients. | *InSight*, a machine learning-based sepsis-prediction algorithm with gradient tree boosting. | Retrospective observational multi-center and research database study |
| McCoy et al [43] | 2017 | USA | The study included all patients aged ≥ 18 years in the ICU, PCU, 2East and 4East (cycle 1) and the ED (additionally in cycle 2).  1328 cases, with the pre-implementation period consisting of 407 cases ( November 1, 2016 to January 31, 2017) and two post-implementation periods consisting of 336 cases ( February 7 to March 14, 2017) and 381 cases (March 15 2017 to April 14, 2017), as well as 204 cases in the post-implementation steady-state period (April 20 to May 20, 2017), beginning 1 month after completion of alert modifications. | Cape Regional Medical Center (CRMC), a 242-bed acute care community hospital (Cape May Courthouse, New Jersey, USA). | Retrospective data from November 1, 2016 to January 31, 2017, from the CRMC electronic health record systems: the Allscripts electronic health record (San Jose, California, USA) for emergency department patients and the Cerner Soarian electronic health record system (Kansas City, Missouri, USA) for intensive care unit, progressive care unit and medical/surgical patients (2East and 4East units) was used as baseline measurements. | Machine Learning algorithm developed by *Dascena Inc* (Hayward, California, USA). | Prospective single-center quality improvement study |
| Ocampo-Quintero et al [44] | 2022 | Spain | Does not apply. | Does not apply. | Does not apply. | Does not apply. | Narrative review |
| Rogers et al [45] | 2023 | USA | Does not apply. | Does not apply. | Medicare claims data from 670 severe sepsis and septic shock patients without sepsis-related DRG (870, 871, 872), aged ≥ 18 years, between October 2016 and July 2020. | *COMPOSER (COnformal Multidimensional Prediction Of Sepsis Risk)*, a feedforward neural network followed by a logistic regression developed by Shashikumar et a. [1]. | Retrospective observational single-center study |
| Roggeveen et al [46] | 2021 | The Nether-lands | Does not apply. | Does not apply. | Data from the MIMIC III v1.4 (contains 61532 admissions to the intensive care unit from 2001–2012 from the Beth Israel Deaconess Medical Center in Boston, Massachusetts, USA) database for development of the MLA and data from the AmsterdamUMCdb (contains 23556 intensive care unit admissions from 2003–2016 in Amsterdam UMC, Amsterdam, the Netherlands). database for external validation of the derived model.  The model was developed on septic patients from 24 hours prior to the onset of sepsis up to 48 h hours after the onset of sepsis. | Reinforcement learning model architecture based on a Dueling Double-Deep Q Network (DQN for brevity). | Retrospective observational multi-research database study |
| Scherer et al [47] | 2022 | Brazil | Does not apply. | Does not apply. | Hospitalized patients aged ≥ 18 years were included.  A total of 122703 alarms were extracted from the platform from a reference hospital complex in Porto Alegre, Rio Grande do Sul, Brazil. After filtering exclusion criteria 254 alerts (0.2%) were delimited for 61 inpatients. | *Robot Laura*, using two machine learning algorithms: Support Vector machines and Artificial Neural Networks. | Retrospective observational single-center cohort study |
| Schinkel et al [48] | 2019 | The Nether-lands | Does not apply. | Does not apply. | Does not apply. | Does not apply. | Narrative review |
| Shimabukuro et al [49] | 2017 | USA | From December 2016 to February 2017 all patients aged ≥ 18 years admitted to the participating units were automatically enrolled.  142 patients from the participating units met the inclusion criteria: 75 were assigned to the control group and 67 assigned to the experimental group. | 2 mixed medical-surgical intensive care units at the University of California Medical Center at Parnassus Heights, San Francisco, California, USA. | No information provided. | Prediction MLA developed by *Dascena Inc* (Hayward, California, USA). | Single-center randomized clinical trial |
| Voermans et al [50] | 2019 | The Nether-lands | Does not apply. | Does not apply. | Data were collected in 2, 4-year time periods: 2006–2009 (no PCT testing) and after implementation of PCT testing, 2010–2014, in the Five Rivers Medical Center (FRMC), Pocahontas, Arkansas, USA.  Where unavailable, data from Mewes et al 2019 [2] were included. | Decision tree model developed by Kip et al 2015 [3]. | Retrospective observational single-center study |
| Wu M et al [51] | 2021 | China | Does not apply. | Does not apply. | Does not apply. | Does not apply. | Narrative Review |
| **Citation Searching** | | | | | | | |
| Burdick et al [52] | 2018 | USA | All patients over the age of 18 admitted to the emergency department or intensive care units were monitored during the study.  Pre-implementation data: Collected during the period July 1 to July 30, 2017.  Post-implementation data: Collected during the period from August 1 to August 30, 2017.  2296 sepsis-related cases, (1160 patients in the pre-implementation phase and 1136 patients in the post-implementation phase). | Cabell Huntington Hospital (CHH), a 303-bed facility. | No information provided. | *Dascena inc MLA* (machine learning algodiagnostic). | Prospective single-center before-and-after study |
| Henry et al [53] | 2015 | USA | Does not apply. | No information provided. | MIMIC-II Clinical Database.  **Development set:**  13014 adult patients (1836 positive, 11178 negative).  **Validation set:**  Data from 3011 adult patients (455 positive, 2556 negative) in the validation set as though they were observed prospectively. | *TREWScore*, a machine learning model using supervised learning. | Retrospective observational research database study |
| **Institutional Websites** | | | | | | | |
| Hsu et al [54] | 2023 | Taiwan | iAMS is fully implemented in the CMUH since June 2021 and as of the end of 2022, the cumulative times of visits have reached 146,438 with a monthly average of visits of 11,274 in 2022. | China Medical University Hospital (CMUH), Taiwan. | All n = 35966 patients aged ≥ 20 years were included who were suspected of having an infection. | Comprehensive Intelligent AntiMicrobial System, providing personalized antibiogram, sepsis and mortality risk prediction and monitoring, multidrug-resistant organisms detection/prediction, and intelligent antibiotic CDSS. | Prospective single-center case study |
| van der Vegt et al [55] | 2023 | Australia | Does not apply. | Does not apply. | Does not apply. | Does not apply. | Systematic Review |
| Sepsis Alliance [56] | 2021 | USA | No information provided. | Sepsis Alliance Clinical Community and Sepsis Alliance Summit. | No information provided. | *Insight*, a *Dascena Inc* machine learning diagnostic algorithm. | Article in website news section |
| HIMSS [57] | 2020 | USA | No information provided. | All UCHealth hospitals with overall 1250 acute care inpatient beds (rollout between May 2019 and March 2020). | No information provided. | MLA (no further information provided). | Article in website news section |

## References

[1] Shashikumar SP, Wardi G, Malhotra A, Nemati S. Artificial intelligence sepsis prediction algorithm learns to say "I don't know". NPJ Digit Med 2021 Sep 09;4(1):134. doi: 10.1038/s41746-021-00504-6

[2] Mewes J, Pulia M, Mansour M, Broyles M, Nguyen H, and Steuten L. The cost impact of PCT-guided antibiotic stewardship versus usual care for hospitalised patients with suspected sepsis or lower respiratory tract infections in the US: A health economic model analysis. PLoS One 2019 Apr 23;14(4):e0214222. doi: 10.1371/journal.pone.0214222

[3] Kip MMA, Kusters R, IJzerman M, and Steuten L. A PCT algorithm for discontinuation of antibiotic therapy is a costeffective way to reduce antibiotic exposure in adult intensive care patients with sepsis. J Med Econ 2015;18(11):944-53. doi: 10.3111/13696998.2015.1064934

1. The country of origin always corresponds to the origin of the first authors institution. [↑](#footnote-ref-1)
2. The study population is only relevant for prospective and qualitative study designs. [↑](#footnote-ref-2)
3. The setting is only relevant for prospective and qualitative study designs. [↑](#footnote-ref-3)
