# Supplementary material for: Patient Benefits in the Context of Sepsis-Related AI-Based Clinical Decision Support Systems: Scoping Review
Source: J Med Internet Res. 2026 Jan 26;28:e76772. doi: 10.2196/76772 (PMC12834200; doi:10.2196/76772)
Supplement: Multimedia Appendix 7 [file jmir-v28-e76772-s007.docx]

# Multimedia Appendix 8. Patient Benefits Mentioned in Articles.

| **Author(s)** | **Prediction** | **Earlier Treatment and Prioritization** | **Individualized Therapy** | **Improved SOFA-Score** | **Reduced Length of Stay** | **Reduced Mortality** | **General Improvements in Care** | **Reduced Readmission Rate** |
| --- | --- | --- | --- | --- | --- | --- | --- | --- |
| **Systematic Research** | | | | | | | | |
| Adams, et al (2022) [28] |  | ● |  | ● | ● | ● |  |  |
| Bologheanu, et al (2023) [29] |  |  | ● |  |  | ● |  |  |
| Bunn, et al (2021) [30] |  |  | ● |  |  |  |  |  |
| Burdick, et al (2020) [31] | ● | ● |  |  | ● | ● | ● | ● |
| Ferreira, et al (2022) [32] |  |  |  |  |  | ● |  |  |
| Garnica, et al (2021) [33] | ● | ● | ● |  |  | ● |  |  |
| Ginestra, et al (2019) [34] |  |  |  |  |  |  | ● |  |
| Goh, et al (2021) [35] | ● |  |  |  |  |  |  |  |
| Henry, et al (2022) [36] |  | ● |  |  |  |  |  |  |
| Joshi, et al (2022) [37] | ● | ● |  |  |  |  |  |  |
| Kausch, et al (2021) [38] | ● |  |  |  |  |  | ● |  |
| Komorowski, et al (2018) [39] |  |  | ● |  |  | ● |  |  |
| Kuo, et al (2021) [40] | ● | ● |  |  |  |  |  |  |
| Ma, et al (2021) [41] |  |  | ● |  |  |  |  |  |
| Mao, et al (2018) [42] | ● |  |  |  |  |  |  |  |
| McCoy, et al (2017) [43] |  |  |  |  | ● | ● | ● | ● |
| Ocampo-Quintero, et al (2022) [44] |  |  | ● |  |  |  |  |  |
| Rogers, et al (2023) [45] | ● |  | ● |  |  |  |  |  |
| Roggeveen, et al (2021) [46] |  |  | ● |  |  |  |  |  |
| Scherer, et al (2022) [47] |  |  | ● |  |  |  | ● |  |
| Schinkel, et al (2019) [48] | ● |  |  |  |  | ● | ● |  |
| Shimabukuro, et al (2017) [49] |  | ● |  |  | ● | ● | ● |  |
| Voermans, et al (2019) [50] |  |  | ● |  | ● |  |  |  |
| Wu M, et al (2021) [51] | ● |  |  |  |  |  |  |  |
| **Citation Searching** | | | | | | | | |
| Burdick, et al (2018) [52] |  | ● |  |  | ● | ● |  |  |
| Henry, et al (2015) [53] | ● | ● |  |  |  |  |  |  |
| **Institutional Websites** | | | | | | | | |
| Hsu, et al (2023) [54] |  |  | ● |  | ● | ● | ● |  |
| Van der Vegt, et al (2023) [55] |  |  |  |  |  | ● |  |  |
| Sepsis Alliance (2021) [56] | ● |  |  |  |  | ● |  |  |
| HIMSS (2020) [57] |  | ● |  |  |  | ● |  |  |
| **Total** | | | | | | | | |
|  | n = 12 | n = 10 | n = 11 | n = 1 | n = 7 | n = 14 | n = 8 | n = 2 |
