# Supplementary material for: Patient Benefits in the Context of Sepsis-Related AI-Based Clinical Decision Support Systems: Scoping Review
Source: J Med Internet Res. 2026 Jan 26;28:e76772. doi: 10.2196/76772 (PMC12834200; doi:10.2196/76772)
Supplement: Multimedia Appendix 8 [file jmir-v28-e76772-s008.docx]

# Multimedia Appendix 9. Screened Articles.

| **No.** | **Author** | **Year** | **Title** | **Decision** | **Reason** |
| --- | --- | --- | --- | --- | --- |
| 1 | Adams, et al. | 2022a | Assessing clinical use and performance of a machine learning sepsis alert for sex and racial bias | No | E5: Conference abstract |
| 2 | Adams, et al. | 2022b | Prospective, multi-site study of patient outcomes after implementation of the TREWS machine learning-based early warning system for sepsis | Yes |  |
| 3 | Agor, Li, Özaltin | 2022 | Septic shock prediction and knowledge discovery through temporal pattern mining | No | Not available |
| 4 | Alam, Rahmani | 2023 | FedSepsis: A Federated Multi-Modal Deep Learning-Based Internet of Medical Things Application for Early Detection of Sepsis from Electronic Health Records Using Raspberry Pi and Jetson Nano Devices | No | E4: Not addressing research questions in more detail |
| 5 | Arriaga-Pizano, et al. | 2021 | Accurate diagnosis of sepsis using a neural network: Pilot study using routine clinical variables | No | E4: Not addressing research questions in more detail |
| 6 | Aşuroğlu, Oğul | 2021 | A deep learning approach for sepsis monitoring via severity score estimation | No | E2: Focus on description of binary classifiers |
| 7 | Back, et al. | 2016 | Development and Validation of an Automated Sepsis Risk Assessment System | No | E2: Focus on description of binary classifiers |
| 8 | Barton, et al. | 2018 | Effect of a machine learning-based severe sepsis prediction algorithm on patient survival | No | E5: Conference abstract |
| 9 | Bhargava, et al. | 2022 | Assessment of a combined biomarker-EMR data machine learning model for sepsis-3 | No | E5: Conference abstract |
| 10 | Böck, et al. | 2022 | Superhuman performance on sepsis MIMIC-III data by distributional reinforcement learning | No | E1: Exclusively technical description of systems |
| 11 | Bologheanu, et al. | 2023 | Development of a Reinforcement Learning Algorithm to Optimize Corticosteroid Therapy in Critically Ill Patients with Sepsis | Yes |  |
| 12 | Brant, et al. | 2021 | Machine learning-guided early treatment of sepsis | No | E5: Conference abstract |
| 13 | Bunn, et al. | 2021 | Application of machine learning to the prediction of postoperative sepsis after appendectomy | Yes |  |
| 14 | Burdick, et al. | 2020 | Effect of a sepsis prediction algorithm on patient mortality, length of stay and readmission: a prospective multicentre clinical outcomes evaluation of real-world patient data from US hospitals | Yes |  |
| 15 | Carey, et al. | 2022 | Fair Reinforcement Learning for Maternal Sepsis Treatment | No | E4: Not addressing research questions in more detail |
| 16 | Dadwal, et al. | 2018 | A dynamic machine-learning based prediction model for sepsis in patients undergoing hematopoietic stem cell transplantation | No | E5: Conference abstract |
| 17 | Demirer, Demirer | 2019 | Early Prediction of Sepsis from Clinical Data Using Artificial Intelligence | No | E1: Exclusively technical description of systems |
| 18 | Do, et al. | 2021 | Combining Reinforcement Learning with Supervised Learning for Sepsis Treatment | No | E4: Not addressing research questions in more detail |
| 19 | Ericson, et al. | 2022 | The Potential Cost and Cost-effectiveness Impact of Using a Machine Learning Algorithm for Early Detection of Sepsis in Intensive Care Units in Sweden | No | E4: Not addressing research questions in more detail |
| 20 | Falini, et al. | 2019 | Patterns of modified shock indices between survivors and nonsurvivors across cohorts of similar vasopressor equivalents | No | E5: Conference abstract |
| 21 | Ferreira, et al. | 2022 | Using machine learning for process improvement in sepsis management | Yes |  |
| 22 | Festor, et al. | 2022 | Assuring the safety of AI-based clinical decision support systems: A case study of the AI Clinician for sepsis treatment | No | I2 not met: No CDSS |
| 23 | Fukuchi, et al. | 2022 | Optimal sedation strategy for ventilated patients with sepsis using deep reinforcement learning | No | E5: Conference abstract |
| 24 | Gallant, et al. | 2018 | Predicting severe sepsis from the electronic health record using machine learning | No | Not available |
| 25 | Gamboa-Antiñolo | 2021 | Prognostic tools for elderly patients with sepsis: in search of new predictive models | No | I2 not met: No AI |
| 26 | Garnica, et al. | 2021 | Diagnosing hospital bacteraemia in the framework of predictive, preventive and personalised medicine using electronic health records and machine learning classifiers | Yes |  |
| 27 | Giacobbe, et al. | 2021 | Early Detection of Sepsis With Machine Learning Techniques: A Brief Clinical Perspective | No | E4: Not addressing research questions in more detail |
| 28 | Giannini, et al. | 2019 | A Machine Learning Algorithm to Predict Severe Sepsis and Septic Shock: Development, Implementation, and Impact on Clinical Practice | No | E4: Not addressing research questions in more detail |
| 29 | Ginestra, et al. | 2019 | Clinician Perception of a Machine Learning–Based Early Warning System Designed to Predict Severe Sepsis and Septic Shock | Yes |  |
| 30 | Goh, et al. | 2021 | Artificial intelligence in sepsis early prediction and diagnosis using unstructured data in healthcare | Yes |  |
| 31 | Goh, et al. | 2022 | Predicting Bacteremia among Septic Patients Based on ED Information by Machine Learning Methods: A Comparative Study | No | E4: Not addressing research questions in more detail |
| 32 | Gonçalves, et al. | 2014 | Real-Time Predictive Analytics for Sepsis Level and Therapeutic Plans in Intensive Care Medicine | No | E4: Not addressing research questions in more detail |
| 33 | Habli, et al. | 2020 | Artificial intelligence in health care: Accountability and safety | No | I2: not met: No patient benefits mentioned |
| 34 | Henry, et al. | 2022 | Factors driving provider adoption of the TREWS machine learning-based early warning system and its effects on sepsis treatment timing | Yes |  |
| 35 | Henry, et al. | 2018 | Automatically identifying sepsis in retrospective data with a clinical phenotyping algorithm | No | E5: Conference abstract |
| 36 | Hjelmgren, et al. | 2022 | EE550 The Potential Cost and Cost-Effectiveness Impact of Using Machine Learning Sepsis Prediction Algorithm for Early Detection of Sepsis in Intensive Care Units in Sweden and the United Kingdom | No | E5: Conference abstract |
| 37 | Holder, et al. | 2021 | A Locally Optimized Data-Driven Tool to Predict Sepsis-Associated Vasopressor Use in the ICU | No | E2: Focus on description of binary classifiers |
| 38 | Horng, et al. | 2012 | Machine learning algorithms can identify patients who will benefit from targeted sepsis decision support | No | E5: Conference abstract |
| 39 | Humphries, et al. | 2020 | A 29 messenger RNA host response signature identifies bacterial and viral infections among emergency department patients | No | E5: Conference abstract |
| 40 | Javan, et al. | 2019 | An intelligent warning model for early prediction of cardiac arrest in sepsis patients | No | E2: Focus on description of binary classifiers |
| 41 | Jeter, et al. | 2019 | Automated pressure regulation system for sepsis (auto-press): Reinforcement learning agent learns to provide vasopressor and intravenous fluid recommendations to manage hypotensive episodes in septic patients | No | Not available |
| 42 | Jia, et al. | 2020 | Safe Reinforcement Learning for Sepsis Treatment | No | E4: Not addressing research questions in more detail |
| 43 | Joshi, et al. | 2020 | Machine learning-based and rule-based sepsis risk prediction tools: A qualitative study of implementation challenges and approaches | No | E5: Conference abstract |
| 44 | Joshi, et al. | 2022 | Implementation approaches and barriers for rule-based and machine learning-based sepsis risk prediction tools: A qualitative study | Yes |  |
| 45 | Ju, et al. | 2021 | To Reduce Healthcare Workload: Identify Critical Sepsis Progression Moments through Deep Reinforcement Learning | No | E4: Not addressing research questions in more detail |
| 46 | Kamaleswaran, et al. | 2019 | Predicting early post-operative sepsis in liver transplantation applying artificial intelligence | No | E5: Conference abstract |
| 47 | Kanjilal, et al. | 2022 | Antimicrobial stewardship for empirical treatment of bloodstream infection using machine learning clinical decision support | No | E5: Conference abstract |
| 48 | Kausch, et al. | 2021 | Physiological machine learning models for prediction of sepsis in hospitalized adults: An integrative review | Yes |  |
| 49 | Khalili, et al. | 2013 | Using predictive models for clinical decision support | No | Not available |
| 50 | Kheterpal, et al. | 2022 | Digitising the prediction and management of sepsis | No | I2: not met: No patient benefits mentioned |
| 51 | Khoshnevisan, et al. | 2021 | Unifying Domain Adaptation and Domain Generalization for Robust Prediction Across Minority Racial Groups | No | I2: not met: No patient benefits mentioned |
| 52 | Khoshnevisan, et al. | 2018 | Recent Temporal Pattern Mining for Septic Shock Early Prediction | No | E4: Not addressing research questions in more detail |
| 53 | Ko, et al. | 2015 | What was old is new again: Using the host response to diagnose infectious disease | No | I2 not met: No AI and no CDSS |
| 54 | Komorowski | 2019 | Improving sepsis resuscitation with reinforcement learning | No | E5: Conference abstract |
| 55 | Komorowski, et al. | 2018 | The Artificial Intelligence Clinician learns optimal treatment strategies for sepsis in intensive care | Yes |  |
| 56 | Kopanista, et al. | 2021 | Identification of Risk Factors and Prediction of Sepsis in Pregnancy Using Machine Learning Methods | No | E4: Not addressing research questions in more detail |
| 57 | Kuo, et al. | 2021 | Applying artificial neural network for early detection of sepsis with intentionally preserved highly missing real-world data for simulating clinical situation | Yes |  |
| 58 | Lauritsen, et al. | 2020 | Early detection of sepsis utilizing deep learning on electronic health record event sequences | No | I2: not met: No patient benefits mentioned |
| 59 | Lauritsen, et al. | 2021 | The Framing of machine learning risk prediction models illustrated by evaluation of sepsis in general wards | No | E4: Not addressing research questions in more detail |
| 60 | Lin, et al. | 2021 | Machine learning model to identify sepsis patients in the emergency department: Algorithm development and validation | No | I2: not met: No patient benefits mentioned |
| 61 | Linnen, et al. | 2020 | Postimplementation Evaluation of a Machine Learning-Based Deterioration Risk Alert to Enhance Sepsis Outcome Improvements | No | E4: Not addressing research questions in more detail |
| 62 | Liu S, et al. | 2019 | Early Prediction of Sepsis via SMOTE Upsampling and Mutual Information Based Downsampling | No | E2: Focus on description of binary classifiers |
| 63 | Liu Z, et al. | 2021 | HeMA: A hierarchically enriched machine learning approach for managing false alarms in real time: A sepsis prediction case study | No | I2: not met: No patient benefits mentioned |
| 64 | Liu Z, et al. | 2022 | A Machine Learning–Enabled Partially Observable Markov Decision Process Framework for Early Sepsis Prediction | No | Not available |
| 65 | Lu L, et al. | 2022 | Safe Reinforcement Learning for Sepsis Treatment | No | E5: Thesis |
| 66 | Lu M, et al. | 2020 | Is Deep Reinforcement Learning Ready for Practical Applications in Healthcare? A Sensitivity Analysis of Duel-DDQN for Hemodynamic Management in Sepsis Patients | No | E4: Not addressing research questions in more detail |
| 67 | Lv, et al. | 2022 | Account of Deep Learning-Based Ultrasonic Image Feature in the Diagnosis of Severe Sepsis Complicated with Acute Kidney Injury | No | E2: Focus on description of binary classifiers |
| 68 | Ma, et al. | 2021 | Individualized resuscitation strategy for septic shock formalized by finite mixture modeling and dynamic treatment regimen | Yes |  |
| 69 | Madushani, et al. | 2022 | Early Biomarker Signatures in Surgical Sepsis | No | I2 not met: No CDSS |
| 70 | Manetti, et al. | 2022 | Adoption of novel biomarker test parameters with machine learning-based algorithms for the early detection of sepsis in hospital practice | No | I2: not met: No patient benefits mentioned |
| 71 | Mao, et al. | 2018 | Multicentre validation of a sepsis prediction algorithm using only vital sign data in the emergency department, general ward and ICU | Yes |  |
| 72 | Mayhew, et al. | 2018 | Flexible, cluster-based analysis of the electronic medical record of sepsis with composite mixture models | No | E4: Not addressing research questions in more detail |
| 73 | McCoy, et al. | 2017 | Reducing patient mortality, length of stay and readmissions through machine learning-based sepsis prediction in the emergency department, intensive care unit and hospital floor units | Yes |  |
| 74 | Mollura, et al. | 2022 | A Reinforcement Learning Application for Optimal Fluid and Vasopressor Interventions in Septic ICU Patients | No | E4: Not addressing research questions in more detail |
| 75 | Moor, et al. | 2021 | Early Prediction of Sepsis in the ICU Using Machine Learning: A Systematic Review | No | E4: Not addressing research questions in more detail |
| 76 | Murugesan, et al. | 2019 | Interpretation of Artificial Intelligence Algorithms in the Prediction of Sepsis | No | E4: Not addressing research questions in more detail |
| 77 | Niemantsverdriet, et al. | 2022a | Transportability and Implementation Challenges of Early Warning Scores for Septic Shock in the ICU: A Perspective on the TREWScore | No | I2: not met: No patient benefits mentioned |
| 78 | Niemantsverdriet, et al. | 2022b | A machine learning approach using endpoint adjudication committee labels for the identification of sepsis predictors at the emergency department | No | E4: Not addressing research questions in more detail |
| 79 | Ocampo-Quintero, et al. | 2022 | Enhancing sepsis management through machine learning techniques: A review | Yes |  |
| 80 | Oh, et al. | 2022 | Use of a Policy Tree Algorithm to Identify Maximal Treatment Effect of Crystalloid Therapy in a Cohort of Critically Ill Patients With Sepsis | No | Not available |
| 81 | Pan, et al. | 2022 | Using reinforcement learning to establish a prediction model of precise fluid therapy for patients with sepsis | No | E7: No English or German |
| 82 | Pappada, et al. | 2015 | Investigating a novel sepsis risk index for use in an intelligent antibiotic decision support system | No | E5: Conference abstract |
| 83 | Parente, et al. | 2021 | High Inter-Patient Variability in Sepsis Evolution: A Hidden Markov Model Analysis | No | E4: Not addressing research questions in more detail |
| 84 | Park, et al. | 2020 | Continuous detection and prediction model of bacteremia for in-patients: deep learning for time-series EHR data | No | Not available |
| 85 | Parra-Rodriguez, et al. | 2022 | Antibiotic Decision-Making in the ICU | No | E4: Not addressing research questions in more detail |
| 86 | Paxton, et al. | 2013 | Developing predictive models using electronic medical records: challenges and pitfalls | No | I2: not met: No patient benefits mentioned |
| 87 | Peng, et al. | 2018 | Improving Sepsis Treatment Strategies by Combining Deep and Kernel-Based Reinforcement Learning | No | E4: Not addressing research questions in more detail |
| 88 | Petersen, et al. | 2019 | Deep Reinforcement Learning and Simulation as a Path Toward Precision Medicine | No | I2 not met: No CDSS |
| 89 | Rahmani, et al. | 2022 | Assessing the effects of data drift on the performance of machine learning models used in clinical sepsis prediction | No | I2: not met: No patient benefits mentioned |
| 90 | Rajendran, et al. | 2022 | Data Heterogeneity in Federated Learning with Electronic Health Records: Case Studies of Risk Prediction for Acute Kidney Injury and Sepsis Diseases in Critical Care | No | I2: not met: No patient benefits mentioned |
| 91 | Reyna, et al. | 2020 | Early prediction of sepsis from clinical data: The PhysioNet/computing in cardiology challenge 2019 | No | E4: Not addressing research questions in more detail |
| 92 | Rogers, et al. | 2022 | Optimizing the Implementation of Clinical Predictive Models to Minimize National Costs: Sepsis Case Study | Yes |  |
| 93 | Roggeveen, et al. | 2021 | Transatlantic transferability of a new reinforcement learning model for optimizing haemodynamic treatment for critically ill patients with sepsis | Yes |  |
| 94 | Roggeveen, et al. | 2022 | Right dose, right now: bedside, real-time, data-driven, and personalised antibiotic dosing in critically ill patients with sepsis or septic shock—a two-centre randomised clinical trial | No | E4: Not addressing research questions in more detail |
| 95 | Sandhu, et al. | 2020 | Integrating a machine learning system into clinical workflows: Qualitative study | No | I2: not met: No patient benefits mentioned |
| 96 | Scherer, et al. | 2022 | Beyond technology: Can artificial intelligence support clinical decisions in the prediction of sepsis? | Yes |  |
| 97 | Schinkel, et al. | 2019 | Clinical applications of artificial intelligence in sepsis: A narrative review | Yes |  |
| 98 | Sendak, et al. | 2020a | The human body is a black box: supporting clinical decision-making with deep learning | No | I2: not met: No patient benefits mentioned |
| 99 | Sendak, et al. | 2020b | Real-World Integration of a Sepsis Deep Learning Technology Into Routine Clinical Care: Implementation Study | No | E4: Not addressing research questions in more detail |
| 100 | Shashikumar, et al. | 2021a | Domain adaptation for robust predictive modeling across racial groups | No | E5: Conference (poster) abstract |
| 101 | Shashikumar, et al. | 2021b | DeepAISE – An interpretable and recurrent neural survival model for early prediction of sepsis | No | E4: Not addressing research questions in more detail |
| 102 | Sherwin, et al. | 2017 | Performance of a novel computer-based clinical decision support alert and the impact of patient partitioning and optimization to identify septic patients in an urban emergency department | No | E5: Conference abstract |
| 103 | Shickel | 2019 | DeepSOFA: A Continuous Acuity Score for Critically Ill Patients using Clinically Interpretable Deep Learning | No | I1 not met: no focus on sepsis |
| 104 | Shimabukuro, et al. | 2017 | Effect of a machine learning-based severe sepsis prediction algorithm on patient survival and hospital length of stay: A randomised clinical trial | Yes |  |
| 105 | Shrestha, et al. | 2021 | Supervised machine learning for early predicting the sepsis patient: modified mean imputation and modified chi-square feature selection | No | E4: Not addressing research questions in more detail |
| 106 | Singh, et al. | 2021 | On Missingness Features in Machine Learning Models for Critical Care: Observational Study | No | E4: Not addressing research questions in more detail |
| 107 | Soleimani, et al. | 2018 | Early intervention to reduce need for mechanical ventilation in sepsis patients with respiratory failure in the emergency department | No | E5: Conference abstract |
| 108 | Su L, et al. | 2022 | Establishment and Implementation of Potential Fluid Therapy Balance Strategies for ICU Sepsis Patients Based on Reinforcement Learning | No | E4: Not addressing research questions in more detail |
| 109 | Su Q, et al. | 2022 | Advances on machine learning applications in sepsis associated-acute kidney injury | No | E7: No English or German |
| 110 | Suzhen, et al. | 2022 | Construction of a predictive model for early acute kidney injury risk in intensive care unit septic shock patients based on machine learning | No | E7: No English or German |
| 111 | Tang S, et al. | 2021 | Model Selection for Offline Reinforcement Learning: Practical Considerations for Healthcare Settings | No | E4: Not addressing research questions in more detail |
| 112 | Tang Y, et al. | 2019 | Reduced Rank Least Squares for Real-Time Short Term Estimation of Mean Arterial Blood Pressure in Septic Patients Receiving Norepinephrine | No | E4: Not addressing research questions in more detail |
| 113 | Tang Y, et al. | 2021 | Physiology-Informed Real-Time Mean Arterial Blood Pressure Learning and Prediction for Septic Patients Receiving Norepinephrine | No | E1: Exclusively technical description of systems |
| 114 | Teng, et al. | 2020 | A Review of Predictive Analytics Solutions for Sepsis Patients | No | E4: Not addressing research questions in more detail |
| 115 | Teredesai, et al. | 2022 | Sub-Sequence Graph Representation Learning on High Variability Data for Dynamic Risk Prediction in Critical Care | No | I2: not met: No patient benefits mentioned |
| 116 | Theiling, et al. | 2019 | 2 Sepsis Watch: A Successful Deployment of a Deep Learning Sepsis Detection and Treatment Platform | No | E5: Conference abstract |
| 117 | Topiwala, etl al. | 2019 | Examining the clinical utility of insight: A machine-learning approach to sepsis identification | No | E5: Conference abstract |
| 118 | Tsoukalas, et al. | 2015 | From data to optimal decision making: a data-driven, probabilistic machine learning approach to decision support for patients with sepsis | No | E4: Not addressing research questions in more detail |
| 119 | Vellido, et al. | 2018 | Machine learning in critical care: State-of-the-art and a sepsis case study | No | E4: Not addressing research questions in more detail |
| 120 | Voermans, et al. | 2019 | Cost-Effectiveness Analysis of a Procalcitonin-Guided Decision Algorithm for Antibiotic Stewardship Using Real-World U.S. Hospital Data | Yes |  |
| 121 | Wang H, et al. | 2022 | Comparison between machine learning methods for mortality prediction for sepsis patients with different social determinants | No | I2: not met: No patient benefits mentioned |
| 122 | Wang Zeyu, et al. | 2022 | Learning Optimal Treatment Strategies for Sepsis Using Offline Reinforcement Learning in Continuous Space | No | E4: Not addressing research questions in more detail |
| 123 | Wang Zijie J, et al. | 2022 | Interpretability, Then What? Editing Machine Learning Models to Reflect Human Knowledge and Values | No | E4: Not addressing research questions in more detail |
| 124 | Wardi, et al. | 2020 | Predicting Progression to Septic Shock in the Emergency Department Using an Externally Generalizable Machine-Learning Algorithm | No | E2: Focus on description of binary classifiers |
| 125 | Wei, et al. | 2022 | Research progress on application of artificial intelligence in early diagnosis and prediction of sepsis | No | E7: No English or German |
| 126 | Winterbottom | 2023 | FOCUS ON INPATIENT SEPSIS: ARTIFICIAL INTELLIGENCE, CLINICAL DECISION SUPPORT, AND E-RAPID RESPONSE | No | E5: Conference abstract |
| 127 | Wu M, et al. | 2021 | Artificial Intelligence for Clinical Decision Support in Sepsis | Yes |  |
| 128 | Wu X, et al. | 2023 | A value-based deep reinforcement learning model with human expertise in optimal treatment of sepsis | No | E1: Exclusively technical description of systems |
| 129 | van Wijk, et al. | 2017 | How much data should we collect? A case study in sepsis detection using deep learning | No | E4: Not addressing research questions in more detail |
| 130 | Xia, et al. | 2020 | Prognostic model of small sample critical diseases based on transfer learning | No | E7: No English or German |
| 131 | Yan, et al. | 2022 | Sepsis prediction, early detection, and identification using clinical text for machine learning: a systematic review | No | E4: Not addressing research questions in more detail |
| 132 | Yoo, et al. | 2022 | Development of an Interoperable and Easily Transferable Clinical Decision Support System Deployment Platform: System Design and Development Study | No | I2 not met: No AI |
| 133 | Zhang L, et al. | 2022 | Prediction of prognosis in elderly patients with sepsis based on machine learning (random survival forest) | No | E4: Not addressing research questions in more detail |
| 134 | Zhang Q, et al. | 2023 |  | No | E4: Not addressing research questions in more detail |
| 135 | Zhang S, et al. | 2022 | [Construction of a predictive model for early acute kidney injury risk in intensive care unit septic shock patients based on machine learning] | No | E7: No English or German |
| 136 | Zhang Z, et al. | 2020 | Deep learning-based clustering robustly identified two classes of sepsis with both prognostic and predictive values | No | E4: Not addressing research questions in more detail |
| 137 | Zhang Z, et al. | 2021 | Dynamic programming for solving a simulated clinical scenario of sepsis resuscitation | No | E1: Exclusively technical description of systems |
| 138 | Zhang Z, et al. | 2022 | Effectiveness of automated alerting system compared to usual care for the management of sepsis | No | E4: Not addressing research questions in more detail |
| 139 | Zhao, et al. | 2022 | DEAR: Dual-Level Self-attention GRU for Online Early Prediction of Sepsis | No | E4: Not addressing research questions in more detail |
| 140 | Zhou, et al. | 2021 | OnAI-Comp: An Online AI Experts Competing Framework for Early Sepsis Detection | No | E4: Not addressing research questions in more detail |
| 141 | Zoabi, et al. | 2021 | Predicting bloodstream infection outcome using machine learning | No | E4: Not addressing research questions in more detail |
