# Supplementary material for: Patient Benefits in the Context of Sepsis-Related AI-Based Clinical Decision Support Systems: Scoping Review
Source: J Med Internet Res. 2026 Jan 26;28:e76772. doi: 10.2196/76772 (PMC12834200; doi:10.2196/76772)
Supplement: Multimedia Appendix 9 [file jmir-v28-e76772-s009.docx]

# Multimedia Appendix 10. Used Methods per Benefit Category.

| **Method** | **Prediction** | **Earlier Treatment and Prioritization** | **Individualized Therapy** | **Improved SOFA-Score** | **Reduced Length of Stay** | **Reduced Mortality** | **General Improvements in Care** | **Reduced Readmission Rate** |
| --- | --- | --- | --- | --- | --- | --- | --- | --- |
| Prospective Quantitative Study | ● | ● | ● | ● | ● | ● | ● | ● |
| Retrospective Quantitative Study | ● | ● | ● |  | ● | ● | ● |  |
| Mixed Methods Study |  | ● |  |  |  |  |  |  |
| Qualitative Study | ● | ● |  |  |  |  |  |  |
| Review | ● |  | ● |  |  | ● | ● |  |
| Institutional News Report | ● | ● |  |  |  | ● |  |  |
